# The composition of the global and feature specific cyanobacterial core-genomes

**DOI:** 10.3389/fmicb.2015.00219

**Published:** 2015-03-19

**Authors:** Stefan Simm, Mario Keller, Mario Selymesi, Enrico Schleiff

**Affiliations:** ^1^Department of Biosciences, Molecular Cell Biology of Plants, Goethe UniversityFrankfurt am Main, Germany; ^2^Cluster of Excellence Frankfurt, Goethe UniversityFrankfurt am Main, Germany; ^3^Center of Membrane Proteomics, Goethe UniversityFrankfurt am Main, Germany; ^4^Buchmann Institute of Molecular Life Sciences, Goethe UniversityFrankfurt am Main, Germany

**Keywords:** cyanobacteria, *Anabaena* sp. PCC 7120, core-genome, genotypic and phenotypic differences, ortholog search, comparative genomics

## Abstract

Cyanobacteria are photosynthetic prokaryotes important for many ecosystems with a high potential for biotechnological usage e.g., in the production of bioactive molecules. Either asks for a deep understanding of the functionality of cyanobacteria and their interaction with the environment. This in part can be inferred from the analysis of their genomes or proteomes. Today, many cyanobacterial genomes have been sequenced and annotated. This information can be used to identify biological pathways present in all cyanobacteria as proteins involved in such processes are encoded by a so called core-genome. However, beside identification of fundamental processes, genes specific for certain cyanobacterial features can be identified by a holistic genome analysis as well. We identified 559 genes that define the core-genome of 58 analyzed cyanobacteria, as well as three genes likely to be signature genes for thermophilic and 57 genes likely to be signature genes for heterocyst-forming cyanobacteria. To get insights into cyanobacterial systems for the interaction with the environment we also inspected the diversity of the outer membrane proteome with focus on β-barrel proteins. We observed that most of the transporting outer membrane β-barrel proteins are not globally conserved in the cyanobacterial phylum. In turn, the occurrence of β-barrel proteins shows high strain specificity. The core set of outer membrane proteins globally conserved in cyanobacteria comprises three proteins only, namely the outer membrane β-barrel assembly protein Omp85, the lipid A transfer protein LptD, and an OprB-type porin. Thus, we conclude that cyanobacteria have developed individual strategies for the interaction with the environment, while other intracellular processes like the regulation of the protein homeostasis are globally conserved.

## Introduction

Cyanobacteria are ancient, multifarious, photosynthetic prokaryotes. They are of biotechnological importance and are used for approaches to produce bioactive molecules, biofuels or other energy sources (Jones and Mayfield, 2012; Neilan et al., 2013; Wijffels et al., 2013; Oliver and Atsumi, 2014). In addition, cyanobacteria are considered as model organisms to study general aspects of bacteria and cellular processes. In focus are the analysis of the function and evolution of photosynthetic systems (Shih et al., 2013; Croce and van Amerongen, 2014), nitrogen fixation (Bothe et al., 2010; Zehr, 2011), cell to cell communication (Flores and Herrero, 2010; Hahn and Schleiff, 2014), cell differentiation (Muro-Pastor and Hess, 2012), and cell wall function (Nicolaisen et al., 2009; Singh and Montgomery, 2011) to name just a few examples. However, most of the information was established for selected model cyanobacteria and still need to be generalized.

Aside from being of biotechnological importance, cyanobacteria are part of the phytoplankton (Sommer, 2005), but inhabit a diverse range of environments like rocks, lakes and deserts as well (e.g., Mur et al., 1999). It is estimated that all cyanobacteria on earth reach a total biomass of 10^15^g (Garcia-Pichel et al., 2003), which marks these bacteria as an important component of ecosystems. Moreover, due to their high acclimation capacity in fluctuating environments, some cyanobacterial species are thought to show a higher adaptability to climate changes compared to other species. It is discussed that this can result in overgrowing other phytoplankton species within the communities (Carey et al., 2012; Elliott, 2012). The latter requires an efficient uptake of nutrients as well as efficient mechanisms to compete for trace elements. The uptake of solutes depends on outer membrane proteins (OMP; Mirus et al., 2010). Most OMPs are β-barrel proteins, which act in the recognition and transport of solutes, metabolites and proteins (e.g., Nicolaisen et al., 2009; Mirus et al., 2010). Such β-barrel proteins are characteristic for the outer membrane of Gram-negative bacteria, mitochondria and chloroplasts (Sommer et al., 2011). While the transporters of the inner membrane were studied in some detail, not much, however, is known about the existence and function of the outer membrane β-barrel proteins of cyanobacteria (Hahn and Schleiff, 2014).

One measure to generalize the findings and to learn more about cyanobacteria is the pan- and core-genome determination. The pan-genome describes the entire gene set composed of all genes of all strains analyzed (Medini et al., 2005; Collingro et al., 2011). Therefore, it can be determined for an entire phylum like the cyanobacterial phylum (spelled in capitals below to emphasize that the entire phylum is analyzed: PAN-GENOME), or for a reduced set of organisms within the cyanobacterial phylum (spelled in small letters below to indicate that only a part of the PAN-GENOME is assigned: pan-genome). A pan-genome includes a core-genome, a dispensable-genome as well as unique genes (Reno et al., 2009). The dispensable-genome is the set of genes, which occurs in an intersection of at least two, but not all analyzed genomes. Unique genes are found in a single genome only. The core-genome includes those sets of genes that exist in each of the strains analyzed (Kettler et al., 2007). Again, we use capital letters (CORE-GENOME) in case the whole phylum is analyzed and small letters (core-genome) for the analysis of selected cyanobacteria only.

The selection of a subset of strains (clade) for core- and pan-genome analysis can be based on their phylogenetic positioning according to 16S rRNA sequence analysis (e.g., Valério et al., 2009) or traditional morphological features (e.g., Komárek and Anagnostidis, 1986, 1989; Anagnostidis and Komárek, 1987, 1990). In addition, classification of cyanobacteria with respect to their growth habitat offers the opportunity to determine feature-specific sets of genes. The prerequisite for this classification is the definition of morphological, biochemical and physiological features as well as of the typical growth habitat for each strain. Most of this information is deposited in the Integrated Microbial Genomes database (Markowitz et al., 2012). Based on this information, and refined by an exhaustive literature search, we classified the cyanobacterial strains according to 13 distinct features (Table 1, Additional File 1 in Supplementary Material).

**Table 1:** **Phenotypical, ecological and physiological features analyzed**.

	**Feature**	**Sub-categories**	**CWI**
1	Habitat	Sea/Ground/Fresh water/Salt meadow/Host/Water surface/Coast/Mud/Hot spring	56
2	Occurence	Lab/Nature	42
3	Nitrogen fixation	Yes or No	29
4	Toxin production and export	Yes or No	14
5	Trichome	Yes or No	52
6	Cell composition	Unicellular/Filament/Chain/Pairs	56
7	Cell shape	Spherical/Filamentous/Helical/Coccoid/Rod shaped/Oval	52
8	Heterocyst	Yes or No	54
9	Hormogonia	Yes or No	6
10	Akinete	Yes or No	7
11	Temperature range	Mesophilic/Thermophilic	56
12	Oxygen demand	Aerobic/Anaerobic/Facultative aerobic	47
13	Motility	Mobile/Immobile	51

Given is the number (column 1) and name of the feature analyzed (column 2), the categories of the feature (column 3), and the number of cyanobacteria with known information on the specific feature (CWI, column 4). Detailed information are given in Additional File 1 in Supplementary Material.

Previous studies of gene sets have focused on the identification of intra-species gene sets needed to fully describe a species (Medini et al., 2005). The pan-genome analysis was developed as a consequence of the expanding number of sequenced genomes (Medini et al., 2005; Tettelin et al., 2008). Subsequently, this analysis was applied to study single genera like *Prochlorococcus* (Kettler et al., 2007), *Legionella* (D'Auria et al., 2010), or *Streptococcus* (Donati et al., 2010). Today, pan-genome analysis is used to define core-genomes for model organisms like human (Li et al., 2010) or yeast (Dunn et al., 2012). Similarly, core-genome definition of inter-species comparisons in a single phylum was used to gain information on sequence similarity (Tettelin et al., 2005), phylogenetic relations (Kettler et al., 2007) or evolutionary relations, as for example in *Chlamydiae* (Collingro et al., 2011) or cyanobacteria (Beck et al., 2012). Based on core-genome determination for a specific clade of species, the term “signature genes” has been introduced to denote genes with a limited phylogenetic distribution (Dutilh et al., 2008). Core-genome and signature gene definition was used to define a set of genes specific for cyanobacteria against eucaryotes containing chloroplasts (Martin et al., 2003) or specific for the various clades of cyanobacteria (Gupta and Mathews, 2010). This approach has contributed to our knowledge on the origin of photosynthesis (Mulkidjanian et al., 2006) and diversity of metabolism (Beck et al., 2012).

Interestingly, pan- and core-genome analysis was not used to identify feature-specific gene sets yet. Therefore, we investigated gene sets for specific features based on 58 cyanobacterial genomes. We confirmed that the selected genomes are sufficient to define the cyanobacterial CORE-GENOME. In addition, for each genome we determined the genes part of the dispensable-genome and unique genes. Subsequently, cyanobacteria were clustered according to their sequence or feature similarities and we defined the pan- and core-genomes of different clades. This analysis yielded the identification of some genes specific for thermophilic cyanobacteria and for heterocyst forming cyanobacteria. To study the conservation and diversity of the outer membrane proteome, we developed a method for identification of genes coding for β-barrel proteins. The majority of OMPs identified in the PAN-GENOME is not present in the CORE-GENOME. The core-set of β-barrel OMPs in all 58 cyanobacteria is composed of only three proteins, while the majority of the β-barrel OMPs is strain-specific or shared by a small fraction of up to 15 cyanobacteria only. We conclude that the outer membrane proteome is largely adapted to the individual live style and environment of each cyanobacterial strain.

## Materials and methods

### Ortholog search and pan-genome construction

Literature and databases were searched for completely sequenced cyanobacterial genomes or assembled drafts. The respective literature is cited in the Section Introduction. Cyanobacterial nucleotide and protein sequences and other relevant information was taken from Cyanobase (Nakao et al., 2010) and the Integrated Microbial Genomes database of the Joint Genome Institute (Markowitz et al., 2012). The ORFs for each strain were categorized in known and hypothetical based on the deposited description. For the construction of the PAN- and CORE-GENOME, the dispensable-genome and the unique genes we used the complete proteomes of all 58 cyanobacteria. We used OrthoMCL (Chen et al., 2006) for prediction of CLiques of Orthologous Genes (CLOGs). OrthoMCL excluded poor-quality sequences with a length below 10 amino acids or a stop codon frequency higher than 20%. By this approach, all CLOGs containing at least two sequences were detected. Sequences not assigned to a cluster by OrthoMCL were subsequently determined as single-sequence clusters (CLOGs of unique genes).

CLOGs defined by OrthoMCL were evaluated by the Pan-Genome Analysis Pipeline (PGAP) to construct CLOGs of different orders containing more than one strain in their respective orthologous groups (Zhao et al., 2012). The PGAP implemented algorithm used (–method MP) is based on the combination of InParanoid and MultiParanoid (Ostlund et al., 2010). The input files of PGAP had to fulfill the following criteria: (i) a 3:1 relation between the CoDing Sequence (CDS) and protein sequence length had to exist to avoid wrongly annotated protein sequences; (ii) the same amount of CDS to protein sequences for each annotated gene was expected; (iii) the identifier had to be unique. In the end, pan-genomes for Nostocales, Prochlorales, Chroococcales, and Oscillatoriales were created using the parameters for clustering and pan-genome construction (–cluster; –pan-genome). For the PAN-GENOME assignment we used the results of OrthoMCL.

For confirmation of feature specific cyanobacterial signature genes we used all available genomes for Viridiplantae and bacteria (except cyanobacteria) available at NCBI non-redundant (nr) database. We used the sequences of the proteins found in *Thermosynechococcus elongatus* BP-1 (thermophile habitat) or *Anabaena* sp. PCC 7120 (soil living, heterocysts) to blast for similar sequences with at least 80% coverage of the bait sequence and an *e*-value of 1.0 *e*^−10^ or smaller.

To determine the putative function of each CLOG we assigned a functional classification to each sequence of the cyanobacteria (Tatusov et al., 1997) by the Bacterial Annotation System (BASys; van Domselaar et al., 2005) and the information from the WEBserver for Meta-Genome Analysis (WebMGA; Wu et al., 2011).

### Construction of the tanimoto-like index and clustering

The Tanimoto-like index (e.g., Cooper et al., 1993) was used to transform the different features of the cyanobacteria (Additional File 1 in Supplementary Material) in a binary code (bit strings) and calculate the similarity and distance (the latter equals 1-similarity) between two cyanobacteria (Additional File 2 in Supplementary Material). The Tanimoto-like index consists of the sum of bit strings per feature. Each feature may contain more than one subcategory (e.g., habitat: sea, soil, freshwater, host, mud, hot spring, salt marsh) and the amount of subcategories determines the length of each feature bit string. Each subcategory was classified as present (1) or absent (0) based on literature (Additional File 1 in Supplementary Material). Features with no available information were classified as unknown (u). By comparison of two strains we determined whether the feature is (i) unknown in both strains, (ii) known in one strain or (iii) known in both strains. The first case was excluded from further calculations, whereas in the second case the denominator value was increased by 0.5. For the third case we added the sum of ones in the intersection to the numerator and the sum of ones in the union to the denominator (Additional File 2 in Supplementary Material).

### Tree construction

The Tanimoto-like index was used to calculate pair wise distances between strains based on 13 different features (Additional File 3 in Supplementary Material). The distance matrix was used to create the neighbor-joining feature tree (Additional File 4 in Supplementary Material). The CLOG distance neighbor-joining tree (Additional File 4 in Supplementary Material) was based on the CLOG distances (equals 1-similarity) between two strains. The CLOG similarity between two strains was calculated by dividing the number of all shared CLOGs by the number of CLOGs which contained at least one sequence of the two strains. Furthermore, 16S rRNA and average amino acid identity (AAI) neighbor-joining trees were calculated (Additional File 5 in Supplementary Material). The 16S rRNA neighbor-joining tree was based on a multiple alignment via Multiple Alignment using Faster Fourier Transform (MAFFT; Katoh and Standley, 2013). The AAI neighbor-joining tree was built using the 420 CLOGs of the CORE-GENOME that contained one orthologous sequence per strain only. Pairwise global alignments between strains were calculated for each CLOG and the AAI over all CLOGs per pair of strains determined. Neighbor-joining trees were built with the molecular evolutionary genetics analysis package 6 (MEGA6; Tamura et al., 2013). The tree morphology was compared by calculating the patristic distance correlation (between 1 correlation and -1 anti-correlation) using the Mesquite software (Maddison and Maddison, 2011; http://mesquiteproject.org).

### β-barrel protein prediction and clustering

The first step of Trans-Membrane Beta-barrel Prediction (TMBp) was based on the Beta-barrel Outer Membrane protein Predictor (BOMP; Berven et al., 2004), the K-Nearest Neighbor method based predictor (KNN; Hu and Yan, 2008) and the Trans-Membrane Beta-barrel Discriminator (TMBetaDisc; Ou et al., 2008) that are based on physicochemical features and the primary amino acid sequence. The TMBp approach was supported by a program established in our group (Mirus and Schleiff, 2005) in combination with TMHMM (Moller et al., 2001). Sequences detected as β-barrel proteins by more than one predictor were called *probable* β-barrel proteins.

The second step of β-barrel prediction was based on a Profile Hidden Markov Model (pHMM)-approach using the program HMMer (Eddy, 2011). We used the Protein Family (Pfam) database (Finn et al., 2014), OPM (Lomize et al., 2012), OMPdb (Tsirigos et al., 2011), which provide information on domain architecture and structures of β-barrel OMPs to build HMM profiles for each known β-barrel OMP family. These profiles were used to search for β-barrel OMPs in all cyanobacterial proteomes. Protein sequences with at least one detected β-barrel domain were considered as *probable* β-barrel OMP.

In the third step we defined two minor criteria. First, other domains than β-barrel OMP characterizing domains were identified by searching against the complete Pfam database (Finn et al., 2014). A protein was assigned to have the potential to be β-barrel OMP if an amino acid stretch longer than 79 amino acids was not characterized by such a Pfam domain. Secondly, we analyzed the CLOGs containing sequences representing β-barrel OMPs. If more than 50% of all sequences of a CLOG have been assigned as β-barrel OMP by TMBp and pHMM, the assigned proteins were considered as *detected*.

All proteins were subsequently classified (Table 2), namely in proteins detected by all four criteria [category (a)], proteins which fulfill the two main criteria and at least one minor criterion [category (b)], proteins which fulfill the two main criteria only [category (c)] and all other proteins [category (d)]. For all sequences of category (c) we performed *in silico* 3D structure analyzes with Phyre2 (Kelley and Sternberg, 2009). The results were manually inspected resulting in 37 putative β-barrel proteins [category (c); Table 2].

**Table 2:** **β-Barrel probability categorization**.

**Category**	**Major criteria**		**Minor criteria**		***Anabaena* sp. *PCC 7120***	***All cyanobacteria***
	**TMBp**	**pHMM**	**Pfam**	**CLOGs**		
(a)	Probable	Probable	Potential	Detected	39	703
(b)	Probable	Probable	One of the two criteria		7	179
(c)	Probable	Probable	–	–	4^a^/0^b^	78^a^/37^b^
(d)	Others				6089	228,326

Shown is the category of the β-barrel prediction (column 1), the major criteria based on TMBp (column 2) and pHMM (column 3) analysis, the minor criteria based on Pfam search for non-β-barrel domains (column 4) or analysis of the CLOG composition (column 4); the number of identified genes in Anabaena sp. PCC 7120 (column 6) or in all cyanobacteria (column 7).

a before and

b after structural prediction by Phyre2 and manual inspection.

## Results and discussion

### The general composition of cyanobacterial genomes

**Figure 1:**
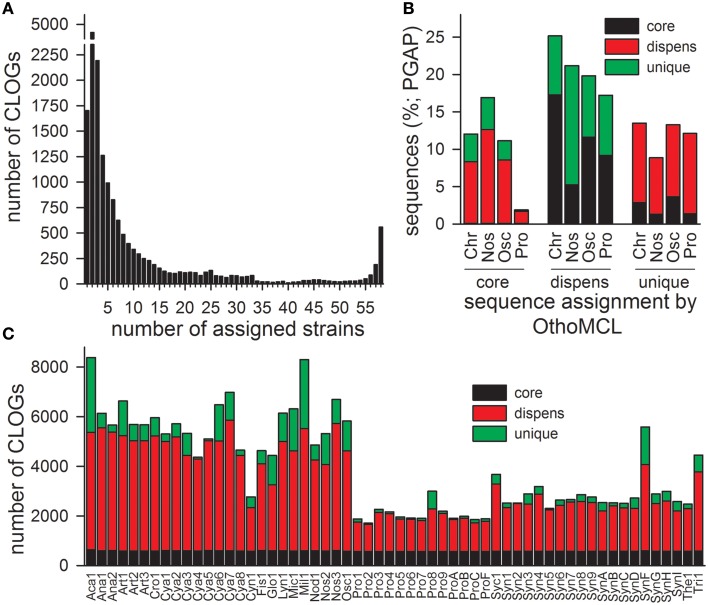
**CLOG distribution of the 58 cyanobacteria. (A)** The numbers cyanobacterial strains from which sequences are included in an individual CLOG was determined. The number of CLOGs containing genes from a given number of strains is shown. **(B)** CLOGs representing unique, dispensable (dispens) or CORE-genes (core) were determined by OrthoMCL for all genomes or by PGAP for the genome of the cyanobacterial order Chroococcales, Nostocales, Oscillatoriales, and Prochlorales. Shown is the frequency of assignment of genes of a certain CLOG category detected by OrthoMCL to another CLOG category by PGAP (CORE-gene: black; dispensable gene: red; unique gene: green). **(C)** Shown is the number of sequences of the individual cyanobacteria represented by a CLOG of the CORE-GENOME (black), by a CLOG of the dispensable-genome (red; dispens), and by a CLOG of unique genes (green).

Sequenced and annotated genomes of 58 cyanobacterial strains representing 45 species from six cyanobacterial orders were used to build the PAN-GENOME (Table 3). We used the amino acid sequences of the proteins encoded by all annotated genes present in the according genome and determined the CLiques of Orthologous Genes (CLOGs). CLOGs with sequences of only one cyanobacterial genome and genes not assigned to any CLOG were classified as “CLOGs of unique genes” for unification of the nomenclature. CLOGs with sequences from a certain set of strains (range from two to 57 strains) were annotated as “CLOGs of the dispensable-genome,” and CLOGs with at least one sequence from each of the 58 strains as “CLOGs of the CORE-GENOME.” We identified 44,831 CLOGs in total. 28,520 of all CLOGs are “CLOGs of unique genes” (Figure 1A). However, it needs to be mentioned that uncertain annotations of hypothetical ORFs can cause a high number of unique genes. Indeed, in Cya7, Cya6, Cya5, Cya4, Cya3, Cya2, Cya1, ProC, Tri1, Mic1, Cya8, Nod1, Glo1 genomes more than 50% of all genes are annotated as “hypothetical”. The outcome of this is that 23,781 “CLOGs of unique genes” are “hypothetical” based on the protein sequence description. Moreover, 1725 of the “CLOGs of unique genes” contain two or more sequences from one strain representing putative paralogs. 15,752 are CLOGs of the dispensable-genome, but most of these CLOGs contain only sequences from up to 10 strains (Figure 1A). Finally, 559 CLOGs of the CORE-GENOME (Additional File 6 in Supplementary Material) were identified as they contain sequences of all 58 cyanobacterial strains (Figure 1A). This is consistent with the earlier postulation that the CORE-GENOME of cyanobacteria has a size of 500–600 genes (Beck et al., 2012).

**Table 3:** **Classification and genome size of the analyzed 58 cyanobacterial strains**.

**Order**	**Species**	**Strain**	**Abbr.**	**Size (Mb)**	**ORFs**	**Put. ORFs (%)**
Chroococcales	Acaryochloris marina	*Acaryochloris marina* MBIC11017	Aca1	8.36	8383	52.75
	*Crocosphaera watsonii*	*Crocosphaera watsonii* WH 8501	Cro1	6.24	5958	44.56
	*Cyanothece* sp. ATCC 51142		Cya1	5.46	5304	56.73
	*Cyanothece* sp. PCC 7424		Cya2	6.55	5710	36.18
	*Cyanothece* sp. PCC 7425		Cya3	5.79	5327	33.40
	*Cyanothece* sp. PCC 8801		Cya4	4.79	4367	29.86
	*Cyanothece* sp. ATCC 51472		Cya5	5.43	5109	31.45
	*Cyanothece* sp. CCY 0110		Cya6	5.88	6475	61.64
	*Cyanothece* sp. PCC 7822		Cya7	7.84	6981	46.48
	*Cyanothece* sp. PCC 8802		Cya8	4.80	4648	34.47
	*Cyanobium* sp. PCC 7001		Cyn1	2.83	2771	32.52
	*Microcystis aeruginosa*	*Microcystis aeruginosa* NIES-843	Mic1	5.84	6311	53.40
	*Synechococcus elongatus*	*Synechococcus elongates* PCC 6301	Syn2	2.70	2525	44.40
		*Synechococcus elongates* PCC 7942	Syn7	2.74	2662	38.92
	*Synechocystis* sp. PCC 6803		Syc1	3.95	3672	50.03
	*Synechococcus* sp. WH 8102		Syn1	2.43	2526	46.00
	*Synechococcus* sp. CC9311		Syn3	2.61	2892	38.00
	*Synechococcus* sp. PCC 7002		Syn4	3.41	3186	31.17
	*Synechococcus* sp. CC9902		Syn5	2.23	2304	39.67
	*Synechococcus* sp. CC9605		Syn6	2.51	2638	45.94
	*Synechococcus* sp. JA-2-3B	*Synechococcus* sp. JA-2-3B'a(2–13)	Syn8	3.05	2862	32.29
	*Synechococcus* sp. JA-3-3Ab		Syn9	2.93	2760	31.88
	*Synechococcus* sp. RCC307		SynA	2.22	2535	36.25
	*Synechococcus* WH7803		SynB	2.37	2533	33.48
	*Synechococcus* sp. BL107		SynC	2.29	2507	44.28
	*Synechococcus* sp. CB0205		SynD	2.43	2719	42.18
	*Synechococcus* sp. PCC 7335		SynF	5.97	5586	45.95
	*Synechococcus* sp. WH 7805		SynG	2.63	2883	49.60
	*Synechococcus* sp. WH 8016		SynH	2.69	2990	35.55
	*Synechococcus* sp. WH 8109		SynI	2.12	2577	39.74
	*Thermosynechococcus elongatus*	*Thermosynechococcus elongatus* BP-1	The1	2.59	2476	42.37
Gloeobacterales	*Gloeobacter violaceus*	*Gloeobacter violaceus* PCC 7421	Glo1	4.66	4431	57.98
Nostocales	*Anabaena* sp. PCC 7120		Ana1	7.21	6135	56.95
	*Anabaena variabilis*	*Anabaena variabilis* ATCC 29413	Ana2	7.11	5661	34.98
	*Nodularia spumigena*	*Nodularia spumigena* CCY9414	Nod1	5.32	4860	50.41
	*Trichormus azollae*	*Nostoc azollae* 0708	Nos2	5.49	5321	60.42
	*Nostoc punctiforme*	*Nostoc punctiforme* PCC 73102	Nos3	9.06	6690	39.07
Oscillatoriales	*Lyngbya* sp. CCY 8106		Lyn1	7.04	6142	53.61
	*Coleofasciculus chthonoplastes*	*Microcoleus chthonoplastes* PCC 7420	Mil1	8.68	8294	57.14
	*Arthrospira platensis*	*Arthrospira platensis* NIES-39	Art1	6.79	6630	61.70
	*Arthrospira maxima*	*Arthrospira maxima* CS-328	Art2	6.00	5690	36.50
	*Arthrospira* sp. PCC 8005		Art3	6.17	5675	46.70
	*Oscillatoria* sp. PCC 6506		Osc1	6.68	5822	53.98
	*Trichodesmium erythraeum*	*Trichodesmium erythraeum* IMS101	Tri1	7.75	4451	39.00
Prochlorales	*Prochlorococcus marinus*	*Prochlorococcus marinus* SS120	Pro1	1.75	1882	27.52
		*Prochlorococcus marinus* MED4	Pro2	1.66	1713	29.83
		*Prochlorococcus marinus* MIT 9313	Pro3	2.41	2267	32.91
		*Prochlorococcus marinus str.* NATL2A	Pro4	1.84	2163	40.41
		*Prochlorococcus marinus str.* MIT 9312	Pro5	1.71	1962	35.68
		*Prochlorococcus marinus str.* AS9601	Pro6	1.67	1921	35.97
		*Prochlorococcus marinus str.* MIT 9515	Pro7	1.70	1906	36.41
		*Prochlorococcus marinus str.* MIT 9303	Pro8	2.68	2997	50.75
		*Prochlorococcus marinus str.* NATL1A	Pro9	1.86	2193	46.69
		*Prochlorococcus marinus str.* MIT 9301	ProA	1.64	1907	35.19
		*Prochlorococcus marinus str.* MIT 9215	ProB	1.74	1983	37.17
		*Prochlorococcus marinus str.* MIT 9211	ProC	1.69	1855	37.20
		*Prochlorococcus marinus str.* MIT 9202	ProF	1.69	1890	33.17
Stigonematales	*Fischerella* sp. JSC-11		Fis1	5.38	4627	27.34

Given is the order (column 1), the species according to NCBI and PATRIC taxonomy (column 2; Wattam et al., 2014) and the strain if not identical with the species (column 3) for each cyanobacteria included in this study. Column 4 gives the abbreviation used in here, column 5 gives the genome size of both, chromosomes and plasmids in megabases (Mb) and column 6 gives the number of protein coding open reading frames (ORFs) on the chromosomes and plasmids. Column 7 gives the percentage of the ORFs only annotated as putative/hypothetical.

The distribution of the sequences in the different CLOG categories is by large comparable to the results of the PGAP analysis, which created individual pan-genomes of different cyanobacterial orders (Figure 1B, Zhao et al., 2012). The discrepancy of about 10% observed by the two approaches is expected, because for CLOG definition by OrthoMCL all genomes were analyzed, while due to computational limitations for the PGAP analysis only the genomes of strains of one order could be used.

With respect to the strains we realized that the majority of the genes of each individual strain was assigned to CLOGs of the dispensable-genome (Figure 1C; red). The total number of genes identified in CLOGs of unique genes varies between the different strains (Figure 1C; green) and is primarily related to the genome size (Table 1). This is expected, because smaller genomes generally code for a lower number of proteins (Table 3) and thus, the portion of the genes found in CLOGs of the CORE-GENOME and of the dispensable-genome is larger. However, this rule does not apply to *Prochlorococcus marinus* strain MIT 9303 (Pro8). Nevertheless, the strain MIT 9303 has the largest genome with most annotated ORFs of all *P. marinus* strains, which might explain the larger portion of unique genes. The “additional” genes in *P. marinus* str. MIT 9303 by large encode proteins with putative functions in membrane synthesis and transport (Kettler et al., 2007), which might hint to specific features of this strain when compared to other strains of *P. marinus.*

Further, exceptions from the rule are *Cyanothece* sp. PCC 8801 (Cya4), *Cyanothece* sp. ATCC 51472 (Cya5) and *Cyanothece* sp. PCC 8802 (Cya8), which have the smallest genome as well as assigned proteome of all *Cyanothece* species (Table 3). These three species show a large content of genes assigned either to the CORE-GENOME or the dispensable-genome, but a small content of unique genes when compared to other *Cyanothece* species. Thus, the genome of these three strains might be composed of genes for the basic functions of *Cyanothece* only.

### The size of the cyanobacterial core- and PAN-genome

**Figure 2:**
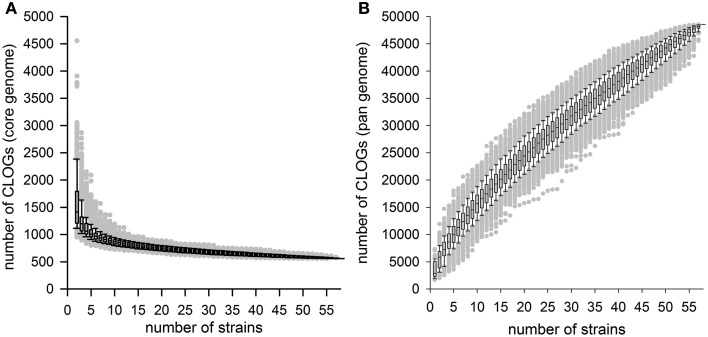
**The cyanobacterial core- and pan-genome**. **(A,B)** The number of CLOGs of the cyanobacterial **(A)** core-genome or **(B)** pan-genome for a given number of organisms is shown. The box plots were created for the results of 1000 different random selections of different cyanobacterial strains. Further simulations are shown in Additional File 6 in Supplementary Material.

Based on the analysis of the 58 cyanobacterial strains a CORE-GENOME size of 559 genes was observed. To judge whether the 45 species represented by the 58 strains are sufficient to define the CORE-GENOME of cyanobacteria, we determined the CORE-GENOME size dependence on the number of genomes analyzed. We determined the size of the core-genome for a given number of randomly selected genomes from the 58 organisms. The random selection was 1000 times repeated and the average calculated (Figure 2A). The number of sequences found in the core-genome changed only little when more than 40 cyanobacterial strains were considered. The result was not dependent on number of repetitions, as for only 100 or even 10,000 random selections the same result was observed (Additional File 7 in Supplementary Material).

The robustness of our result prompted us to compare the CORE-GENOME determined in here with the CORE-GENOMES defined earlier analyzing eight (Martin et al., 2003; 179 CORE-GENES asssigned), 15 (Mulkidjanian et al., 2006; 1044 CORE-GENES asssigned) or 16 cyanobacterial genomes (Beck et al., 2012; 704 CORE-GENES asssigned). The overlap between previously assigned CORE-GENOMES and the one defined in here consists of 520 and 526 sequences for the two larger studies, respectively. On the one hand, this shows that almost all genes of the CORE-GENOME identified in here are present in the previous CORE-GENOME sets, on the other hand it documents that the low number was not sufficient, which is consistent with our simulation (Figure 2A). Both conclusions support the notion that the CORE-GENOME of cyanobacteria most likely covers about 500 genes.

We determined the functional categories based on the sequences of *Anabaena* sp. PCC 7120 for the CORE-GENOME. Here we used the functional annotation previously established for clusters of orthologous groups (COG) for seven complete genomes from five major phylogenetic lineages (Tatusov et al., 1997). In part, the result was manually compared to the KEGG annotations (Kanehisa and Goto, 2000). We realized that proteins encoded by 231 sequences of the CORE-GENOME (representing ~40%) are involved in metabolic processes in *Anabaena* sp. PCC 7120 (Table 4). Thereof, 59 proteins are assigned to be involved in amino acid transport and metabolism (category E), 52 as coenzyme transport and metabolism (category H) and 47 in energy production and conversion (category C). The observation that not all components of the photosystems are encoded by the CORE-GENOME was confirmed by the analysis of the distribution of the proteins involved in oxidative phosphorylation, photosynthesis and antenna proteins annotated by KEGG (Additional File 8 in Supplementary Material). In addition, 90 proteins coded by the CORE-GENOME genes in *Anabaena* sp. PCC 7120 are assigned to be involved in translation, ribosomal structure and biogenesis (category J), while 41 encoded proteins function in posttranslational modification, protein turnover and chaperones and 40 in replication, recombination and repair (Table 4).

**Table 4:** **Functional categories and processes according to COG**.

**Functional category**	**Functional process**	**Abbr.**	**CORE CLOGs**
Information storage and processing	Translation, ribosomal structure and biogenesis	J	90
	Transcription	K	11 (3)^*^
	Replication, recombination and repair	L	37 (3)
	TOTAL		141
Cellular processes and signaling	Cell cycle control, cell division, chromosome partitioning	D	11
	Defense mechanisms	V	1
	Signal transduction mechanisms	T	8
	Cell wall/membrane/envelope biogenesis	M	27
	Cell motility	N	–
	Intracellular trafficking, secretion, and vesicular transport	U	10 (1)
	Posttranslational modification, protein turnover, chaperons	O	40 (1)
	TOTAL		103
Metabolism	Energy production and conversion	C	45 (2)
	Carbohydrate transport and metabolism	G	22 (1)
	Amino acid transport and metabolism	E	49 (10)
	Nucleotide transport and metabolism	F	23 (4)
	Coenzyme transport and metabolism	H	46 (6)
	Lipid transport and metabolism	I	15 (3)
	Inorganic ion transport and metabolism	P	13 (2)
	Secondary metabolites biosynthesis, transport and catabolism	Q	3 (2)
	TOTAL		213
Poorly characterized	General function prediction only	R	35
	Function unknown	S	77
	mixed process^**^	X	17
	TOTAL		129

Given is the global functional category (column 1), the functional process (column 2), the one letter code for the functional process (column 3) and number of proteins per functional assignment of all proteins encoded by the CORE-GENOME of Anabaena sp. PCC 7120. The CLOG annotation is exemplarily for “Energy production and conversion” to the KEGG annotation (Additional File 7 in Supplementary Material).

* The number of proteins in the bracket is the count of proteins assigned to two process (e.g., translation, ribosomal structure and biogenesis and transcription), and the protein is counted for each of the processes.

** The number proteins assigned to more than two process.

Next, we investigated the PAN-GENOME formed by the 44,831 CLOGs observed for the 58 strains defined. Again, we randomly selected the genes of a given number of strains for the determination of the pan-genome and this random selection was repeated 100, 1000, and 10,000 times (Figure 2B; Additional File 7 in Supplementary Material). As for the core-genome analysis, the result was not dependent on the number of random selections used in here. Previously it was postulated that increase of the PAN-GENOME follows the power law with respect to number of genomes included (Tettelin et al., 2008; Figure 2B). For *P. marinus* it was reported that addition of new strains into the analysis would always yield an increase of the pan-genome size (a so called “open pangenome”), however with a low rate (the according factor is α = 0.80 suggesting a low increase of the PAN-GENOME size by addition of the genomic information of an additional strain; Tettelin et al., 2008). For all cyanobacteria we obtained an α of 0.35 ± 0.07. This suggests that the PAN-GENOME of all cyanobacteria is (i) a so called open PAN-GENOME and increases with addition of new cyanobacterial strains, because only for α > 1 a limit exists, and (ii) the PAN-GENOME of all cyanobacteria increases more rapidly by addition of new genomes as the pan-genome for a single species of cyanobacteria like *P. marinus*.

### Habitat specific cyanobacterial proteins

We gathered information about ecological, morphological and physiological features for all analyzed strains from the Integrated Microbial Genomes database of the Joint Genome Institute (Markowitz et al., 2012) and from selected publications (Additional File 1 in Supplementary Material; Huber, 1985; Stal and Krumbein, 1985; Jones, 1992; Cohen et al., 1994; Rouhiainen et al., 1995; Kaneko and Tabata, 1997; Gruber and Bryant, 1998; Nakamura et al., 2002; Zhou and Wolk, 2002; El-Shehawy et al., 2003; Lesser, 2003; Urmeneta et al., 2003; Tuit et al., 2004; Araoz et al., 2005; Allewalt et al., 2006; Dworkin et al., 2006; Su et al., 2006; Takaichi et al., 2006; Gao et al., 2007; Kaneko et al., 2007; Kettler et al., 2007; Kim et al., 2007; Campbell et al., 2008; Stockel et al., 2008; Swingley et al., 2008; Bolhuis et al., 2010; Fujisawa et al., 2010; Mejean et al., 2010; Ran et al., 2010; Scott et al., 2010; Carrieri et al., 2011; Larsson et al., 2011; Ploug et al., 2011; Markowitz et al., 2012; Nguyen et al., 2012; Stewart et al., 2012) and extracted 13 different features (Table 1, Additional file 1 in Supplementary Material). In some cases information was logically assumed. For example, unicellular organisms should not contain features characterizing multicellular cyanobacteria like heterocysts, akinetes or hormogonia.

Next, we determined genes specific for a subset of cyanobacterial strains with either thermophilic character, with common growth environment or the capability to differentiate heterocysts, because for the remaining features the assignment for the cyanobacteria is laregely incomplete (Additional file 1 in Supplementary Material). For the identification of such genes we searched for CLOGs containing exclusively sequences of cyanobacterial strains with a certain feature. Subsequently, only the CLOGs of the latter pool with sequences of all cyanobacterial strains with this feature were selected. In our set of organisms we had three thermophilic cyanobacteria, namely *T. elongatus* BP-1 (The1), *Synechococcus* sp. JA-3-3Ab (Syn9), and *Synechococcus* sp. JA-2-3B'a(2–13) (Syn8). We obtained four CLOGs with genes of these three strains only. In *T. elongatus* BP-1 these genes are tlr0324, tlr0548, tlr0547, and tsr0549 (Nakamura et al., 2002, 2003). The protein tlr0324 putatively contains a DNAJ-domain and is predicted to be a Heat shock protein (HSP), while the proteins encoded by the second gene cluster, tlr0548, tlr0547, and tsr0549, are of unknown function. Next we analyzed whether the identified genes are specific to cyanobacteria by searching for similar sequences in plants and bacteria (see Materials and Methods). Sequences with similarity to tlr0548 have been identified in bacterial strains with extreme habitats of the genus Acidithiobacillus (5) and the species *Haliangium ochraceum* (1), *Halothiobacillus neapolitanus* (1), *Sorangium cellulosum* (2), or *Thiothrix nivea* (1), but not in plants. In turn, we did not identify sequences with similarity to tlr0324, tlr0547, and tsr0549 in the bacterial or plant genomes by the approach applied (see Materials and Methods). Thus, these three genes likely represent “signature genes” for thermophilic cyanobacteria.

With respect to the growth habitat we obtained 34 cyanobacterial strains assigned to live in salt water, 15 in fresh water, three in fresh water as well as on soil, three require a host organism, one is exclusively soil-living and one occurrs in both salt and fresh water (Additional File 1 in Supplementary Material). However, we did not find a CLOG including sequences of all cyanobacteria growing in salt or fresh water. The same holds true for the three host-living cyanobacteria. Thus, either a habitat-specific core-genome does not exist with respect to salt/fresh water and host-living strains, or for some of the strains the assignment found in literature is incomplete.

Five CLOGs for the cyanobacterial strains assigned as capable of soil-living (*Anabaena* sp. PCC 7120, *Anabaena variabilis* ATCC 29413, *Gloeobacter violaceus* PCC 7421, *Nostoc punctiforme* PCC 73102) were identified. We again aimed for confirmation of the specificity of the identified genes for cyanobacteria. However, similar sequences to the identified oxidoreductase (encoded by all0827 in *Anabaena* sp. PCC 7120) was found in many other plant and bacterial genomes. Similarily, sequences with similarity to the protein with similarity to acetyltransferases (encoded by alr3061), the membrane-spanning subunit DevC of the heterocyst-specific ABC transporter (encoded by alr4974) and the six-bladed β-propeller TolB-like domain containing protein (encoded by all0351) were identified in many bacterial genomes. Only for the protein of unknown function encoded by alr7204 sequences with similarity could not be identified in the analyzed eucaryotic or prokaryotic genomes. Summing up, we propose the existence of at least three signature genes for thermophilic and one signature gene for soil-living cyanobacteria, while we could not identify signature genes for salt or fresh water living cyanobacteria.

### Heterocyst specific cyanobacterial proteins

**Figure 3:**
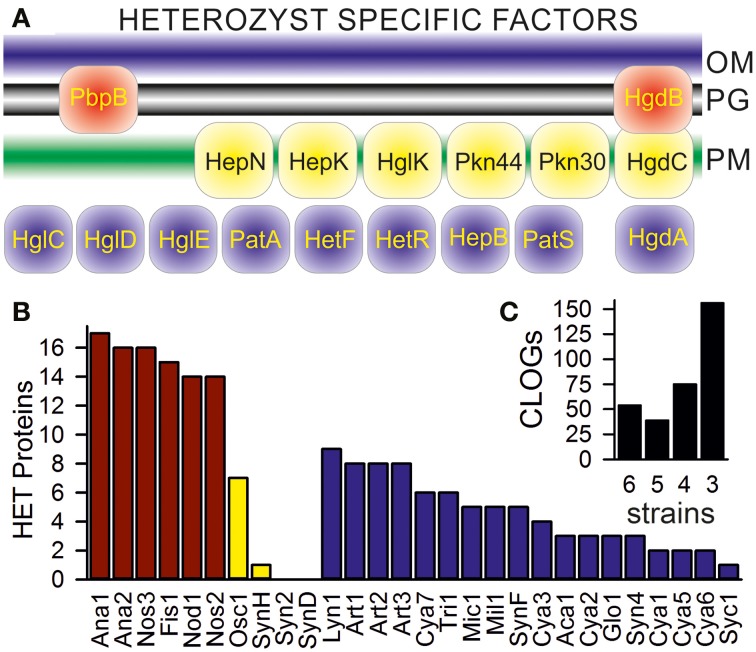
**CLOGs of genes involved in heterocyst formation. (A)** A scheme of the localization of the 17 selected heterocyst specific proteins is shown: the penicillin-binding protein 2 (alr5101 in *Anabaena* PCC 7120, PbpB; Lazaro et al., 2001), the pentapeptide-repeat protein HglK (all0813; Black et al., 1995), the oxidoreductase HgdA (all5345; Nicolaisen et al., 2009), the glycolipid deposition proteins HgdB and HgdC (all5347 and all5346; Nicolaisen et al., 2009), the HstK family proteins with two-component sensor domain Pkn44 and Pkn30 (all1625 and all3691; Shi et al., 2007), the sensory protein-histidine kinase of a two-component regulatory system (all4496; HepK; Golden and Yoon, 2003), the ketoreductase HetN (alr5358; Corrales-Guerrero et al., 2014), the heterocyst differentiation control proteins HetR (alr2339; Du et al., 2012) and HetF (alr3546; Ionescu et al., 2010), the poly-peptides controlling heterocyst pattern formation PatA (all0521; Zhang et al., 2007) and PatS (asl2301; Nicolaisen et al., 2009), the heterocyst envelope polysaccharide synthesis factor HepB (alr3698; Wang et al., 2007) and the heterocyst glycolipid synthases HglC, HglD, and HglE (alr5355, alr5354, and alr5351; Fan et al., 2005). **(B)** CLOGs including *Anabaena* sp. PCC 7120 sequences mentioned in the text have been analyzed concerning the cyanobacteria the sequences originated from. The number of detected proteins known to be involved in heterocyst formation/function in cyanobacteria known to form heterocysts (red), not to form heterocysts (yellow) or for which information about heterocysts formation is not available (blue) is shown. **(C)** The inset on the right shows the number of CLOGs with the sequences of the six heterocyst-forming cyanobacteria only.

We aimed for the detection of CLOGs unifying heterocyst-forming cyanobacteria. In our set six cyanobacteria are assigned as heterocyst-forming (Additional File 1 in Supplementary Material; *Anabaena* sp. PCC 7120, *Anabaena variabilis* ATCC 29413; *Fischerella* sp. JSC-11; *Nodularia spumigena* CCY9414; *Nostoc azollae* 0708; *Nostoc punctiforme* PCC 73102), while for four cyanobacteria information was not available (*Oscillatoria* sp. PCC 6506, *Synechococcus* sp. WH 8016; *Synechococcus elongates* PCC 6301; *Synechococcus* sp. CB0205). To judge whether we have to include the latter four as heterocyst forming, we inspected CLOGs containing genes known to be essential for heterocyst differentiation, but not related to global families like the ABC transporters. We selected 17 of such genes (Figure 3A). Sequences of all confirmed heterocyst-forming cyanobacteria (Additional File 1 in Supplementary Material; Ana1, Ana2, Fis1, Nod1, Nos2, Nos3) are in 14 of the 17 CLOGs formed by the selected heterocyst marker genes (Figure 3B, red bars). Only PatS (asl2301, *Anabaena* sp. PCC 7120), HetN (alr5358, *Anabaena* sp. PCC 7120), and PbpB (alr5101, *Anabaena* sp. PCC 7120) could not be detected in all strains by the method applied.

Nine CLOGs of genes known to be essential to heterocyst differentiation contain sequences of the filamentous *Lyngbya* sp. CCY 8106; and eight CLOGs contain sequences of each of the *Arthrospira* strains, though for these cyanobacteria heterocyst formation is not reported (Figure 3B, blue bars, Additional File 1 in Supplementary Material). These eight CLOGs represent PbpB, HglK, HgdA, HetR, HetF, Pkn44, Pkn30, and HepK. The meaning of this observation needs to be explored in future.

Of the four strains with unknown assignment to heterocyst formation, sequences of the filamentous *Oscillatoria* sp. PCC 6506 are present in seven of the 17 CLOGs of the selected heterocyst specific genes (Figure 3B, yellow bar). As expected sequences of the three most likely unicelluar strains (Syn2, SynD, SynH) are detectable in at most one of the 17 CLOGs (Figure 3B, yellow bar). Consequently, from our inspection of the distribution of genes specific for heterocysts we conclude that only the six confirmed heterocyst forming cyanobacteria should be included in the analysis of the core-genome of genes specific for heterocyst forming cyanobacteria.

At first we identified all CLOGs with sequences from the six heterocyst-forming strains only. We observed 54 CLOGs with sequences from all six strains, 39 with sequences from five, 75 from four and 156 from three heterocyst-forming cyanobacteria (Figure 3C). The number of CLOGs with sequences of only five strains prompted us to consider the 93 genes of the CLOGS containing sequences of at least five of the six strains as core-genome of heterocyst-forming cyanobacteria (Tables 5, 6). Fourteen of these 93 genes have been experimentally charactarized and for 28 a function can be predicted (Table 5), while for 51 genes a function is not assigned (Table 6). Eight of the 93 genes were shown to be exclusively expressed upon nitrogen starvation in *Anabaena* PCC 7120, while another 48 genes are at least two-fold higher expressed either 12 or 21 h after nitrogen step-down (Tables 5, 6, Flaherty et al., 2011). In turn, only one gene is not expressed in *Anabaena* PCC 7120 after nitrogen starvation (asl1933) and one is significantly downregulated (asr1289; Table 5, Flaherty et al., 2011).

**Table 5:** **Genes with known or putative function in heterocyst-specific CLOGs**.

**Acc. Number**	**Name**	**Function**	**FC**		**CA**	**V/B**	**References**
			**12 h**	**21 h**			
all0521	PatA	Heterocyst formation regulating two-component response regulator	1,6	1,4		0/0	Liang et al., 1992
all1866	TrxA2	Thioredoxin A2	2,8	3,7	Fis1	391/499	Ehira and Ohmori, 2012
all2356	PhnE	Phosphonate ABC transport permease	5,9	6,1	Nos2	0/490	Pernil et al., 2010
alr2392	FraC/SepJ	Filament integrity protein	−1,7	1,9		0/0	Bauer et al., 1995
alr2834	HepC	Glycosyl transferase	47,3	19,2		0/0	Zhu et al., 1998
alr2837		Glycosyl transferase of group 2	Up	up		0/27	Huang et al., 2005
alr3234		Similar to heterocyst formation protein HetP	−1,2	−1,3	Fis1	0/0	Higa and Callahan, 2010
alr3287	NrtB	Nitrate transport protein	1,1	1,9	Nod1	0/479	Herrero et al., 2001
alr3732	PknE	Protein serine-threonine kinase	3,8	1,2		0/0	Zhang et al., 1998
alr4368	PknD	Serine/threonine kinase	3,0	1,4		0/0	Zhang and Libs, 1998
all5341	HglT	Glycosyl transferase of group 1	up	up		48/485	Awai and Wolk, 2007
all5344		Unknown	not	up		0/141	Fan et al., 2005
all5346	HgdC	Membrane spanning subunit of heterocyst specific ABC-transporter	not	34,6		0/85	Fan et al., 2005
all5347	HgdB	Membrane fusion protein of heterocyst specific ABC-transporter	2,3	115,8		0/62	Fan et al., 2005
all0059		Lipopolysaccharide biosynthesis protein	53,6	19,2		0/71	None
all1345		Probable glycosyl transferase	−1,2	−1,3		0/185	None
all1862		Putative peptidase	22,2	9,6	Fis1	0/0	None
all2008		Serine proteinase	1,2	1,2		6/198	None
all2068		Alpha/beta hydrolase fold protein	1,3	1,0		59/482	None
all2357		Phosphonate ABC transport ATP-binding component	4,9	3,3	Nos2	485/497	None
all2358		Periplasmic phosphonate binding protein	6,3	2,9		0/148	None
alr2463		Aminoglycoside phosphotransferase	9,8	3,6		0/1^a^	None
alr3125		Heme oxygenase	−2,5	2,4	Nod1	0/385	None
alr3235	TrpC	Indole-3-glycerol phosphate synthase	up	up	Fis1	89/498	None
alr3246		Pyridoxamine 5′ phosphate oxidase Related protein	up	up	Fis1	0/429	None
all3306		Pentapeptide repeat containing protein	up	up	Fis1	0/21	None
all3559		Putative peptidase	−1,7	1,5	Nod1	0/0	None
alr3774		Rhomboid like protein	3,5	2,4		0/419	None
alr3931		Rhomboid family protein	1,1	−1,0	Nos2	0/485	None
alr3948	CbiQ	Cobalt transport protein	6,8	4,2		0/1^b^	None
all3984		Predicted ATP-dependent protease	2,1	1,0		0/0	None
all4051		Prc barrel domain containing protein	2,3	2,7		0/30	None
all4538		Mannose-6-phosphate isomerase	1,5	−1,2		0/107	None
all4729		Putative metalloprotein	−1,0	100,8		0/1^c^	None
asl4754	PetM	Cytochrome b6f complex subunit	−2,5	−1,8		0/0	None
all4768		ErfK/YbiS/YcfS/YnhG family protein	2,7	7,5	Nod1	0/11	None
alr4812	PatN	Heterocyst differentiation related protein	1,3	1,4	Fis1	0/0	None
alr4877		WD40-repeat protein	2,5	2,7	Nod1	0/0	None
alr4898		Transcriptional regulator	2,1	1,6	Fis1	3/90	None
alr4984		Peptidoglycan binding domain 1 containing protein	25,4	5,7		0/1^d^	None
asr5289		Similar to subunit X of photosystem I	1,2	1,0		0/0	None
all5304		Secretion protein HlyD family protein	6,0	3,2		0/491	None
ava0606		Transmembrane protein	not	not	Ana1	0/0	None

Shown is the accession number of Anabaena sp. PCC 7120 or Anabaena variables ATCC 29413; column 1, the name and function of the gene if assigned (column 2, 3), the fold change (FC) of expression after 12 and 21 h of nitrogen starvation compared to 0 h (Flaherty et al., 2011; column 4, 5; up, infinite; not, not expressed), the cyanobacteria for which no sequence is identified in the according CLOG (CA, column 6), the number of sequences found in the genomes of Viridplantae or bacteria (V/B, column 7) and a references for functional relevance for heterocyst function or development (column 8).

a Candidatus Solibacter usitatus.

b Thalassospira profundimaris.

c Rhodopseudomonas palustris.

d Paenibacillus mucilaginosus.

**Table 6:** **Genes of unknown function in heterocyst-specific CLOGs**.

**Acc. number**	**Fold change**		**Cyanob. absent**	**V/B**
	**12 h**	**21 h**		
asl0176	1,9	4,8		0/0
alr0255	8,5	4,9		0/0
all0307	5,8	3,0	Fis1	0/0
asr0460	1,6	not	Nos2	0/0
asr0461	−1,0	−1,9	Nos2	0/0
all0463	7,7	10,6	Nos2	0/0
asr0680	−1,6	−1,9	Fis1	0/19
alr0805	1,4	1,2		2/0^a^
asl0842	−1,5	−1,6	Fis1	0/0
all0997	−4,9	−1,8	Fis1	0/0
alr1137	−1,6	−2,7		0/0
alr1146	9,1	5,3		0/1^b^
alr1147	2,5	1,8	Nos2	0/2^c^
alr1148	8,7	7,7		0/0
asr1289	−2,7	−2,7	Fis1	0/0
all1395	up	up	Nos2	0/0
asl1412	3,6	3,4		0/0
asr1775	1,9	2,2	Nos2	0/0
all1814	15,5	5,9		0/0
asl1933	not	not	Fis1	0/0
all2003	4,0	1,8		0/1^d^
all2089	1,8	1,3	Nos2	0/0
all2344	1,5	−1,1		0/0
alr2366	−1,1	−1,1	Nos2	0/0
alr2374	3,3	2,3		0/0
alr2522	up	up		0/0
asr3134	−1,7	−2,6	Nod1	0/0
asr3279	4,8	7,7	Nos2	0/0
all3520	2,5	2,4	Fis1	0/0
alr3562	1,3	−1,7		0/0
all3568	1,1	−1,0		0/445
all3696	13,2	6,1		0/243
alr3720	9,3	3,6		0/0
all3745	−1,6	−1,6	Fis1	0/0
alr3910	−2,1	−1,5	Nos2	0/0
all4073	4,6	8,5		0/0
asl4098	1,6	1,3		0/0
all4117	2,4	1,8		0/0
all4381	5,5	5,1		0/0
alr4534	1,5	1,2		0/0
all4555	2,2	1,5	Nod1	0/0
asl4565	1,1	2,5		0/0
alr4684	1,6	4,2	Nos2	0/0
alr4714	2,6	1,8		0/0
asl4743	4,1	1,2		0/0
alr4788	1,7	1,7		0/0
asl4860	−1,2	1,7		0/0
all4962	9,7	5,6	Nos2	0/0
alr5005	1,3	1,1		0/0
asr5071	−1,4	1,1		0/0

Shown is the accession number of Anabaena sp. PCC 7120 (column 1), the fold change of expression after 12 and 21 h of nitrogen starvation compared to 0 h (Flaherty et al., 2011; column 2, 3; up, infinite; not, not expressed), the cyanobacteria for which no sequence is identified in the CLOG (column 5) and he number of sequences found in the genomes of Viridplantae or bacteria (V/B, column 7).

a Glycine max, Solanum lycopersicum.

b Streptomyces aurantiacus.

c Frankia sp. EUN1f, Streptomyces aurantiacus.

d Nitrosococcus halophilus.

We inspected whether the genes identified are heterocyst specific signature genes of cyanobacteria. We realized that six of the experimentally characterized genes and eight genes with putative function are indeed specific for cyanobacteria based on our criteria (see Materials and Methods), because sequences with similarity could not be identified in the analyzed plants and bacteria (Table 5). In addition, for four proteins encoded by the genes identified in the CLOGs formed by heterocyst forming cynobacteria only one other bacterial strain containing a similar sequence was detected (Table 5). In addition, for 44 of the not yet characterized factors similar sequences could not be detected in any of the analyzed genomes, while for additional four only one or two sequences with similarity could be detected (Table 6). We therefore propose that eight of the identified genes are highly specific for heterocyst forming cyanobacteria, while 58 represent heterocyst specific cyanobacterial signature genes. It is worth mentioning, the majority thereof have not yet been characterized.

### The composition of the core-genomes of the different clades of cyanobacteria

**Figure 4:**
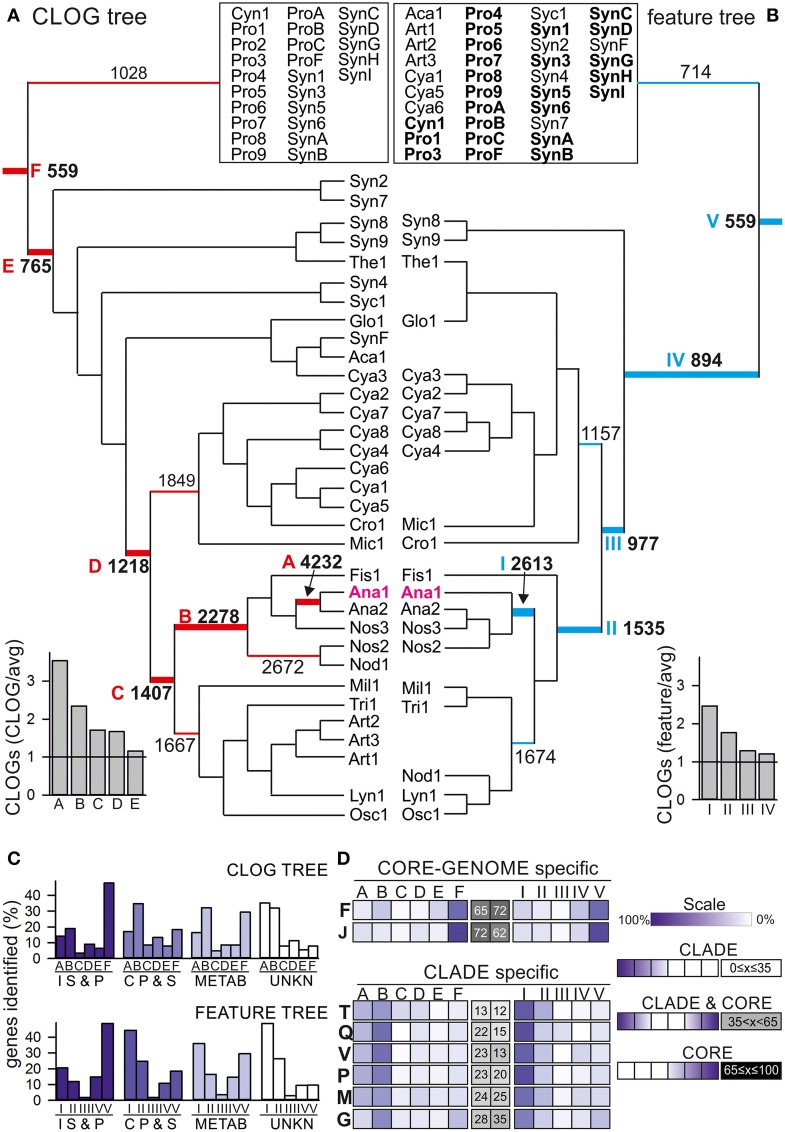
**Feature and shared CLOG correlation tree. (A, B)** The neighbor-joining tree of the 58 cyanobacteria based **(A)** on pair-wise shared CLOGs as distances or **(B)** on the similarities in the 13 selected features as distances was calculated. The root for the different branches from the deepest root (CORE-GENOME) to *Anabaena* sp. PCC 7120 are marked by letter in **A** (F–A) or roman numerals in **B** (I–VI), and the number of CLOGs defining the core-genome for the branch with this root is given. The ratio of the core-genomes of the branches with different roots to the average size of the core-genome expected for this number (Figure 2) is indicated on the bottom left. For simplicity, only branches discussed are shown, while all strains of the remaining part of the tree are clustered in the box on top. The full tree is shown in Additional File 4 in Supplementary Material.**(C)** Each core-genome with the root indicated in **(A,B)** was determined and the number of proteins of a specific category/process (Table 4) additionally found to the core-genome of the deeper roots was counted and is deposited in Additional Files 8, 9 in Supplementary Material. Shown is the occurrence of unique proteins (in percent of all identified proteins) assigned to the four categories “Information storage and processing” (I, S, and P), “Cellular processes and signaling” (C, P, and S), “Metabolism“ (METAB) and unknown (UNKN) in the different clade specific core genomes defined for the CLOG tree (top) and feature tree (bottom). **(D)** Shown is the occurrence of unique proteins assigned to the individual processes (indicated by one letter code shown in Table 4). The distribution for proteins for each process is shown as color code indicated on the right (Scale). For each distribution the profile was analyzed by an inversed gaussian distribution and the position of the minimum was used to assign the process as CLADE specific defined, CLADE and CORE-GENOME defined or CORE genome defined (scale is shown on the right, position of the minimum is given in percent: 0% = exclusive detection in core genome of CLADE A or I, 100% = exclusive detection in CORE-GENOME. The results for equally distributed (CORE and CLADE) genes are shown in Additional File 10 in Supplementary Material.

We calculated a Tanimoto-like index for each pair of cyanobacteria (see Materials and Methods, Additional File 2 in Supplementary Material), which at first transfers the obtained information on cyanobacterial features into a binary code and subsequently determines the similarity of two strains. These indices were used to group the strains (Additional File 3 in Supplementary Material) and to calculate a neighbor-joining tree (Figure 4B, Additional File 4 in Supplementary Material). In parallel, we used the determined CLOGs to calculate the difference between two cyanobacterial strains and used this “pairwise CLOG distance” for calculation of a second neighbor-joining tree (Figure 4A, Additional File 4 in Supplementary Material).

By large, the two trees show a comparable branching (patristic distance correlation coefficient: 0.51). This suggests a correlation between the proteome setup and the analyzed cyanobacterial features. For further verification we compared the CLOG and feature tree with a tree based on the 16S rRNA and the average amino acid identity (AAI) (Additional File 5 in Supplementary Material). As expected, the correlation between CLOG and IAA tree is the highest with a coefficient of 0.83, while the correlation between the feature tree and the two trees was lower but still detectable (correlation of 0.65 and 0.55, respectively). However, some alterations were observed (Figure 4). The CLOG assignment relates the filamentous *Nodularia spumigena* CCY9414 (Nod1) to Nostocales, whereas the feature assignment introduces a shift to Oscillatoriales (Osc1 and Lyn1), because they show similarity in growth habitat, trichome formation and toxin production (Figure 4, Additional File 1 in Supplementary Material). As expected the filamentous *Arthrospira* (Art1–Art3) clustered with Oscillatoriales in the CLOG tree, but not in the feature tree. This shift most likely reflects the assignment of *Arthrospira* as not nitrogen fixing, facultative aerobic, cells with helical cell shape and fresh water living, which is distinct from other Oscillatoriales (Additional File 1 in Supplementary Material). Finally, two Prochlorales strains (*P. marinus* MIT 9313, Pro3; *P. marinus* str. MIT 9303, Pro8) are not assigned to Prochlorales, but to the Chroococcales in the CLOG tree (Additional File 4 in Supplementary Material). For *P. marinus* MIT 9313 which has the second largest genome of all analyzed *P. marinus* strains, we speculate that observed clustering in the CLOG tree results from the large number of genes in “CLOGs of dispensable genes” that contain many genes from other species than *P. marinus* (Figure 1).

We used the two defined trees (Figure 4) to analyze the branch-specific core-genomes with focus on branches including the model system *Anabaena* sp. PCC 7120 (Ana1). At first we compared the size of the core-genomes of the different branches to the expected random average size of core-genomes with the same number of strains (Figure 2A). We realized that the core genome for the strains in clade I (Figure 4A), A and B (Figure 4B) is two-fold larger than expected from our analysis. This could be due the large cyanobacterial genomes in this clade (>5 Mb) when compared to the small genomes from Chroococcales included in the CORE-GENOME calculation. However, this is in agreement with the close relation of the cyanobacteria in these clades. Next, we determined the functional categories based on the sequences of *Anabaena* sp. PCC 7120 for the core-genomes of different branches defined by the indicated roots (Figure 4) of the CLOG (Additional Files 4, 8 in Supplementary Material) and feature-based tree (Additional Files 4, 9 in Supplementary Material) by the strategy described for the CORE-GENOME classification.

We inspected the distribution of the genes of the four functional categories (Figure 4C). For proteins involved in the metabolism (METAB) we found a comparable number in the CORE-GENOME (root F, V; entire tree) as in the clade specific core-genome (root A, B, I, II), while most of the proteins assigned as “Information storage and processing (IS and P)” are found already in the CORE-GENOME (root F, V; Figure 4C). Proteins of unknown function (UNKN) and of “Cellular processes and signaling (CP and S)” are largely found in the clade specific core genomes (root A, B, I, II, Figure 4C). On the one hand this suggests that many strain specific processes have not yet been characterized, on the other hand it can be postulated that cyanobacterial signaling strategies are largely strain specific.

To substantiate the latter notion, we analyzed the distribution of the proteins assigned to the various biological processes (Table 1) in the different clade specific core-genomes. We realized that proteins of most categories are found in the CORE-GENOME of all cyanobacteria as well as in clade specific core genomes (Additional Files 9–11 in Supplementary Material). Only proteins of category N (cell motility) are not represented by the CORE-GENOME, but the detected proteins are equally found in all clade specific core-genomes (Additional Files 11 in Supplementary Material). However, we observed two processes for which most of the proteins are encoded by the CORE-GENOME, namely translation, ribosomal structure and biogenesis (category J), as well as in nucleotide metabolism and transport (category F; Figure 4D). This finding is not unexpected as the process of protein synthesis and nucleotide metabolism were previously identified to be very ancient even existing in the last universal common ancestor (e.g., Poole et al., 1999; Armenta-Medina et al., 2014). In contrast, many proteins classified to be involved in signal transduction and defense mechanisms show a clade specific occurrence (categories V and T, Figure 4D). This supports the above formulated notion that cyanobacterial signaling strategies are largely strain specific.

In addition, proteins involved in inorganic ion, secondary metabolite and carbohydrate metabolism and transport (categories G, P, and Q) as well as in cell wall and cell envelope biogenesis (category M; Figure 4D) are largely CLADE specific. This finding suggests that not only signaling strategies, but also the mechanisms to interact with the environment are specific for small clades of cyanobacteria and even for individual strains.

### The β-barrel proteins in cyanobacteria

To confirm the notion that the proteome for the interaction with the environment, particularly for the uptake and secretion of molecules is highly clade specific, we aimed for the identification of putative OMPs as they are involved in such processes. We focused on proteins characterized by a membrane-embedded β-barrel domain as representative subset of the outer membrane proteome. We developed a consensus approach for the prediction of β-barrel OMPs in the cyanobacterial proteomes (see Materials and Methods). This approach yielded 703 putative β-barrel proteins detected by all criteria [category (a); Table 2], 179 which fulfill the two main criteria and at least one minor criterion [category (b); Table 2] and 37 which fulfill the two main criteria only, but are confirmed by tertiary structure prediction [category (c); Table 2]. All other proteins were not considered as putative β-barrel proteins [category (d); Table 2]. We clustered the sequence stretches representing the putative β-barrel domains of all selected proteins to assign functional properties as previously established (Mirus et al., 2009). We detected 21 clusters of β-barrel proteins with more than four sequences, which represent 12 functional groups based on domains defined by Pfam (Table 7, Additional File 12 in Supplementary Material).

**Table 7:** **Clusters of β-barrel representing sequences**.

**Pfam nomenclature for β-barrel domain**	**Cluster**	**Strain**		Orders^*^		**Sequences**	
(Glucose selective) OprB	9	7	58	2	6	8	295
	20	9		2		15	
	21	58		6		274	
Omp85	10	58		6		155	
LptD (DUF3769)	7	56		6		56	
TonB_dep_Rec/TBDT	6	22		6		124	
OmpA_Pfam/OMPdb	11	14	20	5	5	15	27
	14	3		3		5	
	16	7		2		7	
Omp_β-brl	13	7	17	4	5	10	27
	15	9		1		11	
	18	5		4		6	
DUF3442 Intimin/Invasin	3	5	16	2	3	6	32
	4	3		3		6	
	5	10		2		20	
Fasciclin	17	5		3		5	
DUF481^a^	1	4	15	1	2	6	17
	8	11		2		11	
OmpW	19	5		2		5	
Cellulose synthesis complex barrel/BcsC	2	5		2		5	
Autotransporter	12	4		1		5	

Shown are the names of the Pfam domains characteristic for the β-barrel families (column 1), the number of the cluster according to Additional File 11 in Supplementary Material (column 2), number of strains of which a sequence is present in the cluster (column 3) or in all clusters of the same family (column 4), the number of orders of which sequences are in the cluster (column 5) or in all clusters of the same family (column 6), and the number of different sequences in the cluster (column 7) or in all clusters of the same family (column 8).

* Orders: Chroococcales, Gloeobacterales, Nostocales, Oscillatoriales, Prochlorales, Stigonematales.

a DUF, domain of unknown function.

**Figure 5:**
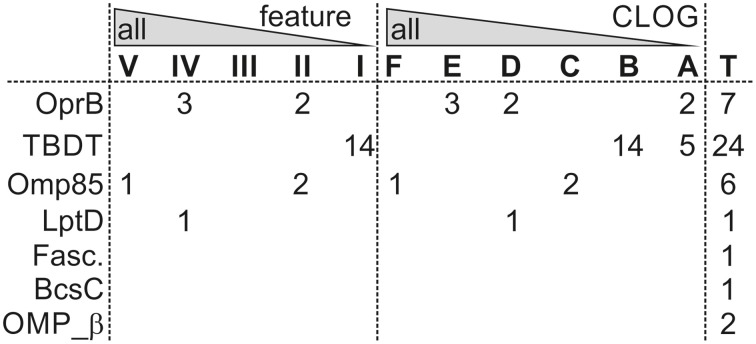
**β-barrel proteins in various core-genomes**. Given are the numbers of OMPs characterized by the indicated domains (Table 7) found in *Anabaena* sp. PCC 7120, which are present in the indicated core-genome of the feature or CLOG tree (Figure 3). T indicates the total number of identified sequences.

Sequences of three β-barrel protein families are found in almost all strains analyzed, namely the OMP of 85 kDa (Omp85; Pfam: Bac_surface_Ag; Moslavac et al., 2005), the lipopolysaccharide transport protein D (LptD; Pfam: DUF3769; Haarmann et al., 2010), and the carbohydrate-selective porin (Pfam: OprB–OMP from *Pseudomonas aeruginosa*; Table 7). Omp85 and LptD are the two central proteins of outer membrane biogenesis of Gram-negative bacteria and belong to the most ancient outer membrane proteins (e.g., Bredemeier et al., 2007; Hahn and Schleiff, 2014), while a porin like OprB is generally required for solute transport. However, only Omp85 is a true component of the CORE-GENOME of cyanobacteria (Figure 5), because orthologs to LptD could not be identified *Acaryochloris marina* and *Synechococcus* sp. *CB0205*, although proteins with low similarity exist. For OprB we realized that the identified sequences cluster in different CLOGs, which is consistent with the detection of the protein family in all strains but the absence in the CORE-GENOME.

In addition, sequences with the broad signature for outer membrane localized β-barrel proteins (OmpA_Pfam/OmpA_OMPdb/OMP-β-brl; cluster 11, 13–16, and 18, Table 7, Additional File 11 in Supplementary Material) are found in the genome of 33 strains of all six cyanobacterial orders, which suggests that most of the cyanobacterial strains have additional outer envelope transporters to OrpB. However, they appear to be strain specific as they are not encoded by any clade specific core genome (Figure 5). The same holds true for the TonB dependent transporter involved in metal transport (Mirus et al., 2009), which was identified in all cyanobacterial orders, but only in 22 strains (Table 7).

All other identified β-barrel protein families are restricted to a lower number of strains and cyanobacterial orders. For example, proteins with a domain characteristic of autotransporters are specific for *Synechococcus* strains (Table 7). Moreover, β-barrel proteins with the INTIMIN/INVASIN domain are only found in five strains of the Prochlorales, in nine *Synechococcus* strains, in *Acaryochloris marina* MBIC11017 and in *Microcoleus chthonoplastes* PCC 7420. Such domains are usually found in virulence factors of enteropathogenic bacteria, mediating invasion into and adherence to host cells (Bodelon et al., 2013). All strains with such proteins are unicellular (except *M. chthonoplastes* PCC 7420) and live in the sea, which might require proteins with such domain for the association of cells to other organisms of the community.

Furthermore, OMPs with a domain characteristic for the cellulose synthase subunit with β-barrel (BcsC) or a FASCLINE domain are found in only eight strains, namely the heterocyst-forming *Anabaena* sp. PCC 7120 (both proteins), *Anabaena variabilis* ATCC 29413 (BcsC), *Nostoc punctiforme* PCC 73102 (both), *Fischerella* sp. JSC-11 (FASCLINE), *Nodularia spumigena* CCY9414 (FASCLINE) as well as in *Acaryochloris marina* MBIC11017 (BscC), *Synechococcus* sp. PCC 7002 (BscC) and *Oscillatoria* sp. PCC 6506 (FASCLINE). BcsC is involved in poly-β-1,6-N-acetyl-D-glucosamine or cellulose export (Keiski et al., 2010). Thus, such a protein might be involved in the formation of the heterocyst specific glycolipid layer and the heterocyst polysaccharide envelope (e.g., Nicolaisen et al., 2009). The FASCICLIN domain is an ancient cell adhesion domain (Borner et al., 2002) that might link the heterocyst specific layer to the outer membrane. In line, the gene of *Anabaena* sp. PCC 7120 (alr3754) with the BscC domain is highly induced (~10-fold) by nitrogen starvation (Flaherty et al., 2011) and the protein with FASCLINE domain was found in heterocyst membrane proteome (Moslavac et al., 2007). Thus, we propose that the function of two OMP families with BcsC or FASCICLIN domains identified in cyanobacteria is most likely related to heterocyst formation, although the experimental evidence is still missing.

From the inspection of the β-barrel proteome we conclude that the basic set for fundamental processes of outer membrane biogenesis represented by Omp85 and LptD and the basic principle of solute exchange represented by OprB are indeed globally conserved, while the majority of the β-barrel OMPs has evolved clade or strain specific to adapt to environmental situations. The large number of proteins with a membrane anchoring domain with general β-barrel signature in various analyzed strains (Table 7: OmpA, Omp_β, DUF481, and OmpW), but with distinct properties leading to a distinct CLOG assignment (Figure 5) supports the above formulated notion that mechanisms to interact with the environment are specific for small clades of cyanobacteria and even for individual strains.

## Conclusion

The analysis of the protein sequences of 58 cyanobacterial strains of six different orders (Table 3) revealed a PAN-GENOME of about 44,831 genes (Figure 2). The cyanobacterial PAN-GENOME is considered to be open, which means that it will increase with each additional genome. In contrast, the CORE-GENOME of the 58 organisms is composed of 559 genes, and it is expected to level off at about 500 sequences (Figure 2). Roughly 20% of the CORE-GENOME is composed of genes involved in protein homeostasis, whereas most of the other genes perform housekeeping functions (Table 4). The individual genomes of cyanobacteria are largely composed of genes of the so-called dispensable-genome genomes, while unique genes are the minority (Figure 1). Based on the comparability of the trees calculated on the base of the genetic information or on features of the cyanobacteria (Figure 3, Table 1) we confirm that features dominate the genomic content. On the one hand, this is supported by the observation that for some features like “heterocyst formation” specific genes can be assigned (Tables 5, 6). On the other hand, analysis of clade specific core-genomes shows the ancient occurrence of processes like translation, ribosomal biogenesis and nucleotide metabolism, while processes involved in reactions to the environment like signal transduction and cell wall biogenesis are highly clade specific (Figure 4). The latter is also supported by the analysis of a specific protein family, namely the β-barrel shaped OMPs. Proteins involved in fundamental processes like outer membrane biogenesis (Omp85, LptD, Figure 5, Table 7) are globally conserved, while the majority of the β-barrel proteins are rather specific for clades of common features or even strain specific (Figure 5). Thus, while the CORE-GENOME describes the housekeeping and protein homeostasis functions, the proteins involved in environment response mechanisms are largely individualized for the various cyanobacteria.

## Author contributions

ES conceptualized, designed and headed the project. SS and MK performed the literature survey, the computational pan-genome and core-genome analysis. SS and MS implemented the β-barrel prediction approach. All authors were involved in analyzing the *in silico* results. ES, MK, and SS were involved in writing the manuscript.

### Conflict of interest statement

The authors declare that the research was conducted in the absence of any commercial or financial relationships that could be construed as a potential conflict of interest.

## Supplementary material

### Supplementary file 1 (Table)—features of the 58 cyanobacterial strains

For all 58 cyanobacteria information on 13 selected features is presented. For better readability, the information is split in three sub-tables. In Tables S1A, S1B the abbreviation assigned to each strain (Table 1) is given (column 1) and the following columns list the information on growth habitat, growth temperature, cultivation in the Lab or collected from nature, cell shape, cell order, mobility and toxin production (Table S1A), as well as on the ability to form Heterocysts, Akinetes, Hormogonia, or Trichome, on the ability to fix nitrogen as well as on their oxygen demand (Table S1B). The source of the information is represented in brackets and the reference is given in Table S1C.

### Supplementary file 2 (Figure)—calculation of the tanimoto-like index

The feature similarity between two strains was calculated by a Tanimoto-like index (see Materials and Methods). Each feature is divided in categories (rectangles) (like feature habitat is divided in the subcategories: mud; fresh water; sea etc.). For each subcategory in each feature a value for present (1), not present (0), or unknown (u) is added. For each feature three different cases could occur: (I) unknown feature in one of two strains (1. feature) counts 0.5 in the denominator, (II) unknown feature of both strains (2. feature) is excluded from counting, and (III) known feature in both strains (3. feature) counts as the quotient of the intersection in the numerator and union in the denominator.

### Supplementary file 3 (Figure)—heat map of feature-based distances of cyanobacteria

Shown is the heat map of the distance of the 58 cyanobacterial strains analyzed in here based on the Tanimoto-like index for the 13 different features. The pair-wise distance is represented in a color code based on percentage calculated by the Tanimoto-like index. Black, 0% distance—related to each other with respect to the features analyzed; white, 100% distance—not related to each other with respect to the features analyzed.

### Supplementary file 4 (Figure)—neighbor-joining trees of Figure 3

(**A, B**)—The neighbor-joining tree of the 58 cyanobacterial organisms is based on their pairwise shared CLOGs (**A**) or the feature distance (**B**). In A the number of shared CLOGs including two organisms is used for distance calculation. In B, the feature distance was calculated by a pairwise Tanimoto-like index based on the intersection of 13 features. The patristic distance correlation had a value of 0.51.

### Supplementary file 5 (Figure)—neighbor-joining trees of 16s rRNA and AAI

**(A, B)**—The neighbor-joining tree of the 58 cyanobacterial strains is based on their alignment of 16S rRNA sequences (**A**) or average amino acid identity (AAI) (**B**). In A the 16S rRNA sequences were multiple aligned by MAFFT. In B, 420 CLOGs of the CORE-GENOME with a single ortholog per strain were pairwise globally aligned the average over the CLOGs calculated to define a distance for each pair of strains. The patristic distance correlation between both trees is 0.76 meaning a strong correlation.

### Supplementary file 6 (Table)—clogs of the core-genome

Shown are the groups of the OrthoMCL ortholog search representing the CLOGs of the CORE-GENOME (column 1) and for each cyanobacterial strain the gene accessions (column 2–59).

### Supplementary file 7 (Figure)—core- and PAN-genome size dependence on the number of analyzed strains

Shown are the numbers of total CLOGs in the core-genome (**A**) or the pan-genome (**B**) derived from the analysis of the given number of organisms (x-axis), which have been randomly selected 100 (left) or 10,000 times (right). The results are plotted as box-plots. Values for 1000 iterations are shown in Figure 2.

### Supplementary file 8 (Table)—distribution of anabaena sp. PCC 7120 proteins involved in oxidative phosphorylation and photosynthesis according to KEGG assignment in the core-genomes of different clades of the feature based tree

The table gives: the root of the clade of the feature based tree for which the core genome was defined (column 1), the KEGG number of the protein (column 2), the name of the protein (column 3), the accession number of the according gene in *Anabaena* sp. PCC 7120 (column 4) and the functional category according to KEGG (column 6) and the functional category according to COG (column 7: Energy prod, energy production and conversion; non, no functional assignment in COG, other, a functional assignment distinct from energy production and conversion).

### Supplementary file 9 (Table)—functional categories of the core-genomes based on the clog-based tree exemplified for anabaena sp. PCC 7120

Given is the functional category (column 1), the abbreviation of the COG of the functional process (column 2) and the number of sequences of *Anabaena* sp. PCC 7120 assigned to the different core-genomes (columns 3–8) based on the CLOG tree (Figure 4A).

### Supplementary file 10 (Table)—functional categories of the core-genomes based on the feature tree exemplified for anabaena sp. PCC 7120

Given is the functional category (column 1), the abbreviation of the COG of the functional process (column 2) and the number of sequences of *Anabaena* sp. PCC 7120 assigned to the different core-genomes (columns 3–8) based on the feature tree (Figure 4B).

### Supplementary file 11 (Figure)—proteins found in core and clade genes

Shown is the occurrence of unique proteins assigned to the individual processes (indicated by one letter code shown in Table 2). The distribution for proteins for each process is shown as color code indicated in Figure 4D. For each distribution the profile was analyzed by an inversed Gaussian distribution and the position of the minimum was used to assign the process as CLADE and CORE-GENOME defined.

### Supplementary file 12 (Figure)—clustering of predicted β-barrel proteins

Shown are clusters of amino acid sequences sections of putative cyanobacterial β-barrel proteins of category (a), (b), and (c) (Table 2) via CLANS. The clusters were numbered and colored according to their predicted function (Table 7). Distances below 1.0 × *e*^−20^ are shown and contain the same functional or domain annotation.
