# Supplementary material for: The composition of the global and feature specific cyanobacterial core-genomes
Source: Front Microbiol. 2015 Mar 19;6:219. doi: 10.3389/fmicb.2015.00219 (PMC4365693; doi:10.3389/fmicb.2015.00219)
Supplement: Supplementary file 1 [file DataSheet1.ZIP › AddFiles/File 1.DOCX]

**Additional file 1: Features extracted from literature for cyanobacterial strains analyzed**

**Table 1A:** We extracted information for each strain with respect to the growth habitat (fresh water, ground, host, sea, cost, mud, salt meadow or water surface), preferred growth temperature (mesophilic, thermophilic), assignments as native collection or laboratory strain (lab, nature), the cell shape (coccoid, oval, helical, rod shaped, spherical, filamentous), the cell order (unicellular, filament, pairs), the mobility (mobile/immobile) and whether they can produce toxins (yes/no). The according literature is cited in the manuscript and listed in Additional Table 1C

| **Strain** | **Habitat** | **Temperature** | **Lab/Nature** | **Cell shape** | **Cell order** | **Mobility** | **Toxine production** |
| --- | --- | --- | --- | --- | --- | --- | --- |
| Aca1 | Sea [1] | Mesophilic [1] | Nature [2] | Spherical [1] | Unicellular [1] | Unknown | Unknown |
| Ana1 | Ground [1] | Mesophilic [1] | Lab | Filamentous [1] | Filament [3] | Mobile [1] | No [4] |
| Ana2 | Ground/Fresh water [1] | Mesophilic [1] | Lab [5] | Filamentous [1] | Unicellular/Filament [1] | Mobile [1] | Unknown |
| Art1 | Fresh water [1] | Mesophilic [1] | Lab [6] | Helical [1] | Filament [7] | Mobile [7] | Unknown |
| Art2 | Fresh water [1] | Mesophilic [1] | Lab | Helical [1] | Filament [7] | Mobile [7] | Unknown |
| Art3 | Fresh water [1] | Mesophilic [1] | Unknown | Helical [1] | Filament [7] | Mobile [7] | Unknown |
| Cro1 | Fresh water [1] | Mesophilic [1] | Lab [1] | Spherical [1] | Unicellular [1] | Unknown | Unknown |
| Cya1 | Sea [1] | Mesophilic [1] | Lab | Coccoid [1] | Unicellular [1] | Immobile [1] | No [web] |
| Cya2 | Fresh water [1] | Mesophilic [1] | Lab | Coccoid [1] | Unicellular [1] | Immobile [1] | No [web] |
| Cya3 | Fresh water [1] | Mesophilic [1] | Lab | Coccoid [1] | Unicellular [1] | Immobile [1] | No [web] |
| Cya4 | Fresh water [1] | Mesophilic [1] | Lab | Coccoid [1] | Unicellular [1] | Immobile [1] | No [web] |
| Cya5 | Sea [1] | Mesophilic [1] | Lab | Coccoid [1] | Unicellular [1] | Immobile [1] | No [web] |
| Cya6 | Sea [1] | Mesophilic [1] | Lab | Coccoid [1] | Unicellular [1] | Immobile [1] | No [web] |
| Cya7 | Fresh water [1] | Mesophilic [1] | Lab | Coccoid [1] | Unicellular [1] | Immobile [1] | No [web] |
| Cya8 | Fresh water [1] | Mesophilic [1] | Lab | Coccoid [1] | Unicellular [1] | Immobile [1] | No [web] |
| Cyn1 | Sea [1] | Mesophilic [1] | Lab | Coccoid [1] | Unicellular [1] | Mobile [1] | Unknown |
| Fis1 | Unknown | Unknown | Unknown | Unknown | Filament [8] | Unknown | Unknown |
| Glo1 | Ground/Fresh water [1] | Mesophilic [1] | Lab | Rod shaped [1] | Unicellular [9] | Immobile [1] | Unknown |
| Lyn1 | Sea/Fresh water/Salt meadow [1] | Mesophilic [1] | Lab | Unknown | Filament [10] | Unknown | Yes [11] |
| Mic1 | Fresh water/Host [1] | Mesophilic [1] | Lab [12] | Coccoid [1] | Unicellular [1] | Immobile [1] | Yes [12] |
| Mil1 | Sea/Salt meadow [1] | Mesophilic [1] | Lab | Filamentous [1] | Filament [13] | Mobile [1] | Unknown |
| Nod1 | Sea/Water surface [1] | Mesophilic [1] | Lab | Filamentous [1] | Filament [3] | Unknown | Yes [14] |
| Nos2 | Host [1] | Mesophilic [1] | Unknown | Filamentous [1] | Filament [1] | Mobile [1] | Unknown |
| Nos3 | Ground/Fresh water [1] | Mesophilic [1] | Lab | Filamentous [1] | Filament [3] | Mobile [1] | Unknown |
| Osc1 | Unknown | Unknown | Lab | Unknown | Filament [15] | Mobile [1] | Yes [15] |
| Pro1 | Sea [1] | Mesophilic [1] | Unknown | Oval [1] | Unicellular [1] | Immobile [1] | Unknown |
| Pro2 | Sea [1] | Mesophilic [1] | Unknown | Oval [1] | Unicellular [1] | Immobile [1] | Unknown |
| Pro3 | Sea [1] | Mesophilic [1] | Unknown | Oval [1] | Unicellular [1] | Immobile [1] | Unknown |
| Pro4 | Sea [1] | Mesophilic [1] | Nature [16] | Oval [1] | Unicellular [1] | Immobile [1] | Unknown |
| Pro5 | Sea [1] | Mesophilic [1] | Unknown | Oval [1] | Unicellular [1] | Immobile [1] | Unknown |
| Pro6 | Sea [1] | Mesophilic [1] | Nature [16] | Oval [1] | Unicellular [1] | Immobile [1] | Unknown |
| Pro7 | Sea/Water surface [1] | Mesophilic [1] | Nature [16] | Oval [1] | Unicellular [1] | Immobile [1] | Unknown |
| Pro8 | Sea [1] | Mesophilic [1] | Nature [16] | Oval [1] | Unicellular [1] | Immobile [1] | Unknown |
| Pro9 | Sea [1] | Mesophilic [1] | Nature [16] | Oval [1] | Unicellular [1] | Immobile [1] | Unknown |
| ProA | Sea [1] | Mesophilic [1] | Nature [16] | Oval [1] | Unicellular [1] | Immobile [1] | Unknown |
| ProB | Sea [1] | Mesophilic [1] | Nature [16] | Oval [1] | Unicellular [1] | Immobile [1] | Unknown |
| ProC | Sea [1] | Mesophilic [1] | Nature [16] | Oval [1] | Unicellular [1] | Immobile [1] | Unknown |
| ProF | Sea [1] | Mesophilic [1] | Unknown | Oval [1] | Unicellular [1] | Immobile [1] | Unknown |
| Syc1 | Fresh water [1] | Mesophilic [1] | Lab | Coccoid [1] | Unicellular [1] | Mobile [1] | No [29] |
| Syn1 | Sea [1] | Mesophilic [1] | Unknown | Coccoid [1] | Unicellular [1] | Mobile [1] | Unknown |
| Syn2 | Fresh water [1] | Mesophilic [1] | Lab | Rod shaped [1] | Unicellular/Chain [1] | Mobile [1] | Unknown |
| Syn3 | Sea [1] | Mesophilic [1] | Lab | Coccoid [1] | Unicellular [1] | Mobile [1] | Unknown |
| Syn4 | Sea/Mud [1] | Mesophilic [1] | Lab | Coccoid [1] | Unicellular [1] | Mobile [1] | Unknown |
| Syn5 | Sea/Coast [1] | Mesophilic [1] | Lab | Coccoid [1] | Unicellular [1] | Mobile [1] | Unknown |
| Syn6 | Sea [1] | Mesophilic [1] | Lab | Coccoid [1] | Unicellular [1] | Mobile [1] | Unknown |
| Syn7 | Fresh water [1] | Mesophilic [1] | Lab | Coccoid [1] | Unicellular/Pairs [1] | Mobile [1] | Unknown |
| Syn8 | Fresh water/Hot spring [1] | Thermophilic [1] | Nature [17] | Coccoid [1] | Unicellular [1] | Mobile [1] | Unknown |
| Syn9 | Fresh water/Hot spring [1] | Thermophilic [1] | Nature [17] | Coccoid [1] | Unicellular [1] | Mobile [1] | Unknown |
| SynA | Sea [1] | Mesophilic [1] | Lab | Coccoid [1] | Unicellular [1] | Mobile [1] | Unknown |
| SynB | Sea [1] | Mesophilic [1] | Unknown | Coccoid [1] | Unicellular [1] | Mobile [1] | Unknown |
| SynC | Sea [1] | Mesophilic [1] | Unknown | Coccoid [1] | Unicellular [1] | Mobile [1] | Unknown |
| SynD | Sea/Host [1] | Mesophilic [1] | Unknown | Unknown | Unknown | Unknown | Unknown |
| SynF | Sea [1] | Mesophilic [1] | Lab | Coccoid [1] | Unicellular [1] | Mobile [1] | Unknown |
| SynG | Sea [1] | Mesophilic [1] | Unknown | Coccoid [1] | Unicellular [1] | Mobile [1] | Unknown |
| SynH | Sea [1] | Mesophilic [1] | Unknown | Unknown | Unknown | Immobile [1] | Unknown |
| SynI | Sea [1] | Mesophilic [1] | Unknown | Coccoid [1] | Unicellular [1] | Mobile [1] | Unknown |
| The1 | Fresh water/Hot spring [1] | Thermophilic [1] | Lab [18] | Rod shaped [1] | Unicellular [1] | Unknown | Unknown |
| Tri1 | Sea [1] | Mesophilic [1] | Unknown | Filamentous [1] | Filament [1] | Mobile [1] | Unknown |

**Table 1B:** We extracted information for each strain with respect to form heterocysts (yes/no), akinetes (yes/no), hormogonia (yes/no), trichome (yes/no), whether they can fix nitrogen (yes/no), their oxygen demand (aerobic, anaerobic, facultative aerobic). The according literature is cited in the manuscript and listed in Table 1C

| **Strain** | **Heterocysts** | **Akinetes** | **Hormogonia** | **Trichome** | **N2 fixation** | **Oxygen demand** |
| --- | --- | --- | --- | --- | --- | --- |
| Aca1 | No | Unknown | Unknown | No | Unknown | Aerobic [1] |
| Ana1 | Yes [3] | Unknown | Unknown | Yes [19] | Yes [3] | Aerobic [1] |
| Ana2 | Yes [20] | Yes [20] | Yes [21] | Unknown | Yes [3] | Aerobic [1] |
| Art1 | No [7] | Unknown | Unknown | Yes [7] | No [5] | Facultative aerobic [1] |
| Art2 | No [7] | Unknown | Unknown | Yes [7] | No [5] | Facultative aerobic [1] |
| Art3 | No [7] | Unknown | Unknown | Yes [7] | No [5] | Facultative aerobic [1] |
| Cro1 | No | Unknown | Unknown | No | Yes [3] | Unknown |
| Cya1 | No | Unknown | Unknown | No | Yes [3] | Facultative aerobic [1] |
| Cya2 | No | Unknown | Unknown | No | Yes [3] | Anaerobic [1] |
| Cya3 | No | Unknown | Unknown | No | Yes [3] | Anaerobic [1] |
| Cya4 | No | Unknown | Unknown | No | Yes [3] | Aerobic [1] |
| Cya5 | No | Unknown | Unknown | No | Yes [17] | Aerobic [1] |
| Cya6 | No | Unknown | Unknown | No | Yes [3] | Aerobic [1] |
| Cya7 | No | Unknown | Unknown | No | Yes [3] | Aerobic [1] |
| Cya8 | No | Unknown | Unknown | No | Yes [3] | Aerobic [1] |
| Cyn1 | No | Unknown | Unknown | No | Unknown | Aerobic [1] |
| Fis1 | Yes [22] | Unknown | Yes [22] | No | Yes [22] | Unknown |
| Glo1 | No | Unknown | Unknown | No | Unknown | Aerobic [1] |
| Lyn1 | No [10] | Unknown | Unknown | Yes [23] | Yes [3] | Aerobic [1] |
| Mic1 | No | Unknown | Unknown | No | Unknown | Aerobic [1] |
| Mil1 | No [13] | Unknown | Unknown | Yes [23] | Yes [3] | Aerobic [1] |
| Nod1 | Yes [3] | Yes [24] | Unknown | Yes [25] | Yes [3] | Aerobic [1] |
| Nos2 | Yes [26] | Yes [26] | Yes [26] | Unknown | Yes [3] | Aerobic [1] |
| Nos3 | Yes [27] | Yes [27] | Yes [27] | Unknown | Yes [3] | Aerobic [1] |
| Osc1 | Unknown | Unknown | Unknown | Yes [28] | Yes [3] | Aerobic [1] |
| Pro1 | No | Unknown | Unknown | No | Unknown | Unknown |
| Pro2 | No | Unknown | Unknown | No | Unknown | Aerobic [1] |
| Pro3 | No | Unknown | Unknown | No | Unknown | Unknown |
| Pro4 | No | Unknown | Unknown | No | Unknown | Aerobic [1] |
| Pro5 | No | Unknown | Unknown | No | Unknown | Aerobic [1] |
| Pro6 | No | Unknown | Unknown | No | Unknown | Aerobic [1] |
| Pro7 | No | Unknown | Unknown | No | Unknown | Unknown |
| Pro8 | No | Unknown | Unknown | No | Unknown | Unknown |
| Pro9 | No | Unknown | Unknown | No | Unknown | Aerobic [1] |
| ProA | No | Unknown | Unknown | No | Unknown | Aerobic [1] |
| ProB | No | Unknown | Unknown | No | Unknown | Aerobic [1] |
| ProC | No | Unknown | Unknown | No | Unknown | Unknown |
| ProF | No | Unknown | Unknown | No | Unknown | Aerobic [1] |
| Syc1 | No | No [20] | Unknown | No | No [29] | Facultative aerobic [1] |
| Syn1 | No | Unknown | Unknown | No | No [30] | Aerobic [1] |
| Syn2 | Unknown | Unknown | Unknown | Unknown | Unknown | Facultative aerobic [1] |
| Syn3 | No | Unknown | Unknown | No | Unknown | Unknown |
| Syn4 | No [19] | No [31] | No [31] | No | No [32] | Facultative aerobic [1] |
| Syn5 | No | Unknown | Unknown | No | Unknown | Aerobic [1] |
| Syn6 | No | Unknown | Unknown | No | Unknown | Unknown |
| Syn7 | No | Unknown | Unknown | No | No [33] | Facultative aerobic [1] |
| Syn8 | No | Unknown | Unknown | No | Yes [3] | Facultative aerobic [1] |
| Syn9 | No | Unknown | Unknown | No | Yes [3] | Facultative aerobic [1] |
| SynA | No | Unknown | Unknown | No | Unknown | Facultative aerobic [1] |
| SynB | No | Unknown | Unknown | No | Unknown | Unknown |
| SynC | No | Unknown | Unknown | Unknown | Unknown | Facultative aerobic [1] |
| SynD | Unknown | Unknown | Unknown | Unknown | Unknown | Unknown |
| SynF | No | Unknown | Unknown | No | Yes [3] | Facultative aerobic [1] |
| SynG | No | Unknown | Unknown | No | Unknown | Facultative aerobic [1] |
| SynH | Unknown | Unknown | Unknown | Unknown | Unknown | Aerobic [1] |
| SynI | No | Unknown | Unknown | No | Unknown | Facultative aerobic [1] |
| The1 | No | Unknown | Unknown | No | Unknown | Aerobic [1] |
| Tri1 | No [34] | No [34] | No [34] | Yes [10] | Yes [3] | Aerobic [1] |

**Table 1C:** The references given in Tables 1A and 1B. All of the references have been cited in the manuscript.

| **Reference number** | **Reference** |
| --- | --- |
| **[1]** | Markowitz VM, Chen IM, Palaniappan K, Chu K, Szeto E, Grechkin Y, Ratner A, Jacob B, Huang J, Williams P et al: IMG: the Integrated Microbial Genomes database and comparative analysis system. Nucleic acids research 2012, 40(Database issue):D115-122. |
| **[2]** | Swingley WD, Chen M, Cheung PC, Conrad AL, Dejesa LC, Hao J, Honchak BM, Karbach LE, Kurdoglu A, Lahiri S et al: Niche adaptation and genome expansion in the chlorophyll d-producing cyanobacterium Acaryochloris marina. Proceedings of the National Academy of Sciences of the United States of America 2008, 105(6):2005-2010. |
| **[3]** | Larsson J, Nylander JA, Bergman B: Genome fluctuations in cyanobacteria reflect evolutionary, developmental and adaptive traits. BMC evolutionary biology 2011, 11:187. |
| **[4]** | Rouhiainen L, Sivonen K, Buikema WJ, Haselkorn R: Characterization of toxin-producing cyanobacteria by using an oligonucleotide probe containing a tandemly repeated heptamer. Journal of bacteriology 1995, 177(20):6021-6026. |
| **[5]** | Fujisawa T, Narikawa R, Okamoto S, Ehira S, Yoshimura H, Suzuki I, Masuda T, Mochimaru M, Takaichi S, Awai K et al: Genomic structure of an economically important cyanobacterium, Arthrospira (Spirulina) platensis NIES-39. DNA research : an international journal for rapid publication of reports on genes and genomes 2010, 17(2):85-103. |
| **[6]** | Carrieri D, Ananyev G, Lenz O, Bryant DA, Dismukes GC: Contribution of a sodium ion gradient to energy conservation during fermentation in the cyanobacterium Arthrospira (Spirulina) maxima CS-328. Applied and environmental microbiology 2011, 77(20):7185-7194. |
| **[7]** | Kim CJ, Jung YH, Oh HM: Factors indicating culture status during cultivation of Spirulina (Arthrospira) platensis. Journal of microbiology 2007, 45(2):122-127. |
| **[8]** | Dworkin MF, S.: The Prokaryotes: Vol. 3: Archaea. Bacteria: Firmicutes, Actinomycetes, vol. 3: Springer Science & Business Media; 2006. |
| **[9]** | Nguyen TA, Brescic J, Vinyard DJ, Chandrasekar T, Dismukes GC: Identification of an oxygenic reaction center psbADC operon in the cyanobacterium Gloeobacter violaceus PCC 7421. Molecular biology and evolution 2012, 29(1):35-38. |
| **[10]** | Jones K: Diurnal nitrogen fixation in tropical marine cyanobacteria: a comparison between adjacent communities of non-heterocystous Lyngbya sp. and heterocystous Calothrix sp. British Phycological Journal 1992, 27(2):107-118. |
| **[11]** | Araoz R, Nghiem HO, Rippka R, Palibroda N, de Marsac NT, Herdman M: Neurotoxins in axenic oscillatorian cyanobacteria: coexistence of anatoxin-a and homoanatoxin-a determined by ligand-binding assay and GC/MS. Microbiology 2005, 151(Pt 4):1263-1273. |
| **[12]** | Kaneko T, Nakajima N, Okamoto S, Suzuki I, Tanabe Y, Tamaoki M, Nakamura Y, Kasai F, Watanabe A, Kawashima K et al: Complete genomic structure of the bloom-forming toxic cyanobacterium Microcystis aeruginosa NIES-843. DNA research : an international journal for rapid publication of reports on genes and genomes 2007, 14(6):247-256. |
| **[13]** | Bolhuis H, Severin I, Confurius-Guns V, Wollenzien UI, Stal LJ: Horizontal transfer of the nitrogen fixation gene cluster in the cyanobacterium Microcoleus chthonoplastes. The ISME journal 2010, 4(1):121-130. |
| **[14]** | Stewart I, Eaglesham GK, McGregor GB, Chong R, Seawright AA, Wickramasinghe WA, Sadler R, Hunt L, Graham G: First report of a toxic Nodularia spumigena (Nostocales/ Cyanobacteria) bloom in sub-tropical Australia. II. Bioaccumulation of nodularin in isolated populations of mullet (Mugilidae). International journal of environmental research and public health 2012, 9(7):2412-2443. |
| **[15]** | Mejean A, Mazmouz R, Mann S, Calteau A, Medigue C, Ploux O: The genome sequence of the cyanobacterium Oscillatoria sp. PCC 6506 reveals several gene clusters responsible for the biosynthesis of toxins and secondary metabolites. Journal of bacteriology 2010, 192(19):5264-5265. |
| **[16]** | Kettler GC, Martiny AC, Huang K, Zucker J, Coleman ML, Rodrigue S, Chen F, Lapidus A, Ferriera S, Johnson J et al: Patterns and implications of gene gain and loss in the evolution of Prochlorococcus. PLoS genetics 2007, 3(12):e231. |
| **[17]** | Advances in Marine Biology cumulative index volumes 20-44. Advances in marine biology 2003, 45:9-312. |
| **[18]** | Nakamura Y, Kaneko T, Sato S, Ikeuchi M, Katoh H, Sasamoto S, Watanabe A, Iriguchi M, Kawashima K, Kimura T et al: Complete genome structure of the thermophilic cyanobacterium Thermosynechococcus elongatus BP-1 (supplement). DNA research : an international journal for rapid publication of reports on genes and genomes 2002, 9(4):135-148. |
| **[19]** | Gao K, Yu H, Brown MT: Solar PAR and UV radiation affects the physiology and morphology of the cyanobacterium Anabaena sp. PCC 7120. Journal of photochemistry and photobiology B, Biology 2007, 89(2-3):117-124. |
| **[20]** | Zhou R, Wolk CP: Identification of an akinete marker gene in Anabaena variabilis. Journal of bacteriology 2002, 184(9):2529-2532. |
| **[21]** | Takaichi S, Mochimaru M, Maoka T: Presence of free myxol and 4-hydroxymyxol and absence of myxol glycosides in Anabaena variabilis ATCC 29413, and proposal of a biosynthetic pathway of carotenoids. Plant & cell physiology 2006, 47(2):211-216. |
| **[22]** | Campbell EL, Christman H, Meeks JC: DNA microarray comparisons of plant factor- and nitrogen deprivation-induced Hormogonia reveal decision-making transcriptional regulation patterns in Nostoc punctiforme. Journal of bacteriology 2008, 190(22):7382-7391. |
| **[23]** | Urmeneta J, Navarrete A, Huete J, Guerrero R: Isolation and characterization of cyanobacteria from microbial mats of the Ebro Delta, Spain. Current microbiology 2003, 46(3):199-204. |
| **[24]** | Huber AL: Factors Affecting the Germination of Akinetes of Nodularia spumigena (Cyanobacteriaceae). Applied and environmental microbiology 1985, 49(1):73-78. |
| **[25]** | Ploug H, Adam B, Musat N, Kalvelage T, Lavik G, Wolf-Gladrow D, Kuypers MM: Carbon, nitrogen and O(2) fluxes associated with the cyanobacterium Nodularia spumigena in the Baltic Sea. The ISME journal 2011, 5(9):1549-1558. |
| **[26]** | Ran L, Larsson J, Vigil-Stenman T, Nylander JA, Ininbergs K, Zheng WW, Lapidus A, Lowry S, Haselkorn R, Bergman B: Genome erosion in a nitrogen-fixing vertically transmitted endosymbiotic multicellular cyanobacterium. PloS one 2010, 5(7):e11486. |
| **[27]** | Cohen MF, Wallis JG, Campbell EL, Meeks JC: Transposon mutagenesis of Nostoc sp. strain ATCC 29133, a filamentous cyanobacterium with multiple cellular differentiation alternatives. Microbiology 1994, 140 ( Pt 12):3233-3240. |
| **[28]** | Stal LJK, W. E.: Nitrogenase activity in the non-heterocystous cyanobacterium Oscillatoria sp. grown under alternating light-dark cycles. Archives of microbiology 1985, 143(1):67-71. |
| **[29]** | Kaneko T, Tabata S: Complete genome structure of the unicellular cyanobacterium Synechocystis sp. PCC6803. Plant & cell physiology 1997, 38(11):1171-1176. |
| **[30]** | Su Z, Mao F, Dam P, Wu H, Olman V, Paulsen IT, Palenik B, Xu Y: Computational inference and experimental validation of the nitrogen assimilation regulatory network in cyanobacterium Synechococcus sp. WH 8102. Nucleic acids research 2006, 34(3):1050-1065. |
| **[31]** | Gruber TM, Bryant DA: Characterization of the alternative sigma-factors SigD and SigE in Synechococcus sp. strain PCC 7002. SigE is implicated in transcription of post-exponential-phase-specific genes. Archives of microbiology 1998, 169(3):211-219. |
| **[32]** | Scott NL, Xu Y, Shen G, Vuletich DA, Falzone CJ, Li Z, Ludwig M, Pond MP, Preimesberger MR, Bryant DA et al: Functional and structural characterization of the 2/2 hemoglobin from Synechococcus sp. PCC 7002. Biochemistry 2010, 49(33):7000-7011. |
| **[33]** | Stockel J, Welsh EA, Liberton M, Kunnvakkam R, Aurora R, Pakrasi HB: Global transcriptomic analysis of Cyanothece 51142 reveals robust diurnal oscillation of central metabolic processes. Proceedings of the National Academy of Sciences of the United States of America 2008, 105(16):6156-6161. |
| **[34]** | El-Shehawy R, Lugomela C, Ernst A, Bergman B: Diurnal expression of hetR and diazocyte development in the filamentous non-heterocystous cyanobacterium Trichodesmium erythraeum. Microbiology 2003, 149(Pt 5):1139-1146. |
| **[web]** | Bundesamt für Verbraucherschutz und Lebensmittelsicherheit,  http://www.bvl.bund.de/SharedDocs/Downloads/06_Gentechnik/ZKBS/01_Allgemeine_Stellungnahmen_deutsch/02_Bakterien/Cyanobakterien.pdf?__blob=publicationFile&v=2 |
