# Supplementary material for: The composition of the global and feature specific cyanobacterial core-genomes
Source: Front Microbiol. 2015 Mar 19;6:219. doi: 10.3389/fmicb.2015.00219 (PMC4365693; doi:10.3389/fmicb.2015.00219)
Supplement: Supplementary file 1 [file DataSheet1.ZIP › AddFiles/File 10.DOCX]

**Additional file 10: Functional categories of the core-genomes based on the feature tree exemplified for *Anabaena* sp. PCC 7120**

| **Functional category** | **Functional process** | **Root of the clade for which the core-genome is defined** | | | | |
| --- | --- | --- | --- | --- | --- | --- |
|  |  | **I** | **II** | **III** | **IV** | **V** |
| **Information storage and processing** | **J** | 15 | 13 | 4 | 20 | 90 |
|  | **K** | 14 | 9 | 1 | 9 | 11 |
|  | **L** | 27 | 8 | - | 12 | 37 |
|  | **KL** | - | 2 | - | - | 3 |
|  | **JKL** | 1 | 1 | - | - | - |
|  | **Total** | **57** | **33** | **5** | **41** | **141** |
| **Cellular processes and signaling** | **D** | 11 | 8 | - | 1 | 11 |
|  | **M** | 64 | 41 | 7 | 25 | 27 |
|  | **N** | - | 1 | - | - | - |
|  | **O** | 44 | 27 | 2 | 12 | 40 |
|  | **T** | 65 | 28 | 1 | 4 | 8 |
|  | **U** | 25 | 8 | - | 1 | 10 |
|  | **V** | 30 | 11 | - | 8 | 5 |
|  | **MU** | - | - | - | 1 | 1 |
|  | **NT** | 3 | 4 | - | - | - |
|  | **NU** | 4 | 5 | - | 6 | - |
|  | **OU** | - | 4 | - | 1 | 1 |
|  | **NOU** | - | - | - | 1 | - |
|  | **Total** | **246** | **137** | **10** | **60** | **103** |
| **Metabolism** | **C** | 38 | 27 | 7 | 29 | 45 |
|  | **E** | 34 | 26 | 6 | 18 | 49 |
|  | **F** | 9 | 4 | 1 | 11 | 23 |
|  | **G** | 46 | 18 | 5 | 10 | 22 |
|  | **H** | 24 | 15 | 4 | 31 | 46 |
|  | **I** | 13 | 3 | 1 | 5 | 15 |
|  | **P** | 81 | 20 | 1 | 4 | 13 |
|  | **Q** | 25 | 6 | 2 | - | 3 |
|  | **CE** | - | - | - | - | 1 |
|  | **CH** | 1 | 1 | - | - | - |
|  | **CP** | - | - | - | 2 | 1 |
|  | **CQ** | 2 | - | - | 3 | - |
|  | **EF** | - | - | - | - | 3 |
|  | **EG** | - | - | - | - | 1 |
|  | **EH** | 3 | 1 | - | 1 | 5 |
|  | **EP** | - | 3 | - | 1 | - |
|  | **EQ** | - | 2 | - | - | - |
|  | **ER** | - | 1 | - | - | - |
|  | **FP** | - | - | - | - | 1 |
|  | **HI** | - | - | - | - | 1 |
|  | **HQ** | 1 | - | - | - | - |
|  | **IQ** | 3 | 1 | - | - | 2 |
|  | **IR** | - | 1 | - | - | - |
|  | **PQ** | 1 | - | 1 | - | - |
|  | **Total** | **281** | **129** | **28** | **115** | **231** |
| **Poorly characterized** | **R** | 92 | 99 | 10 | 40 | 35 |
|  | **S** | 141 | 67 | 5 | 23 | 24 |
|  | **mixed processes** | 57 | 31 | 5 | 10 | 17 |
|  | **-----** | 399 | 156 | 21 | 65 | 53 |
|  | **Total** | **689** | **353** | **41** | **128** | **129** |
| Shown is the functional category (column 1), the abbreviation of the COG of the functional process (Table 2; column 2) and the number of sequences of *Anabaena* sp. PCC 7120 assigned to the different clade core-genomes (columns 3-8) based on the trait tree (Figure 3 B). Please note, the numbers are accumulative: e.g. in V only the number of genes found in addition to the number found in VI are shown. | | | | | | |
