# Supplementary material for: The composition of the global and feature specific cyanobacterial core-genomes
Source: Front Microbiol. 2015 Mar 19;6:219. doi: 10.3389/fmicb.2015.00219 (PMC4365693; doi:10.3389/fmicb.2015.00219)
Supplement: Supplementary file 1 [file DataSheet1.ZIP › AddFiles/File 11.PDF]

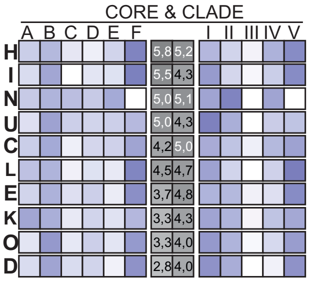
CORE & CLADE
A B C D E F
I II III IV V
H 5,8 5,2
I 5,5 4,3
N 5,0 5,1
U 5,0 4,3
C 4,2 5,0
L 4,5 4,7
E 3,7 4,8
K 3,3 4,3
O 3,3 4,0
D 2,8 4,0
