# Supplementary material for: The composition of the global and feature specific cyanobacterial core-genomes
Source: Front Microbiol. 2015 Mar 19;6:219. doi: 10.3389/fmicb.2015.00219 (PMC4365693; doi:10.3389/fmicb.2015.00219)
Supplement: Supplementary file 1 [file DataSheet1.ZIP › AddFiles/File 5.PDF]

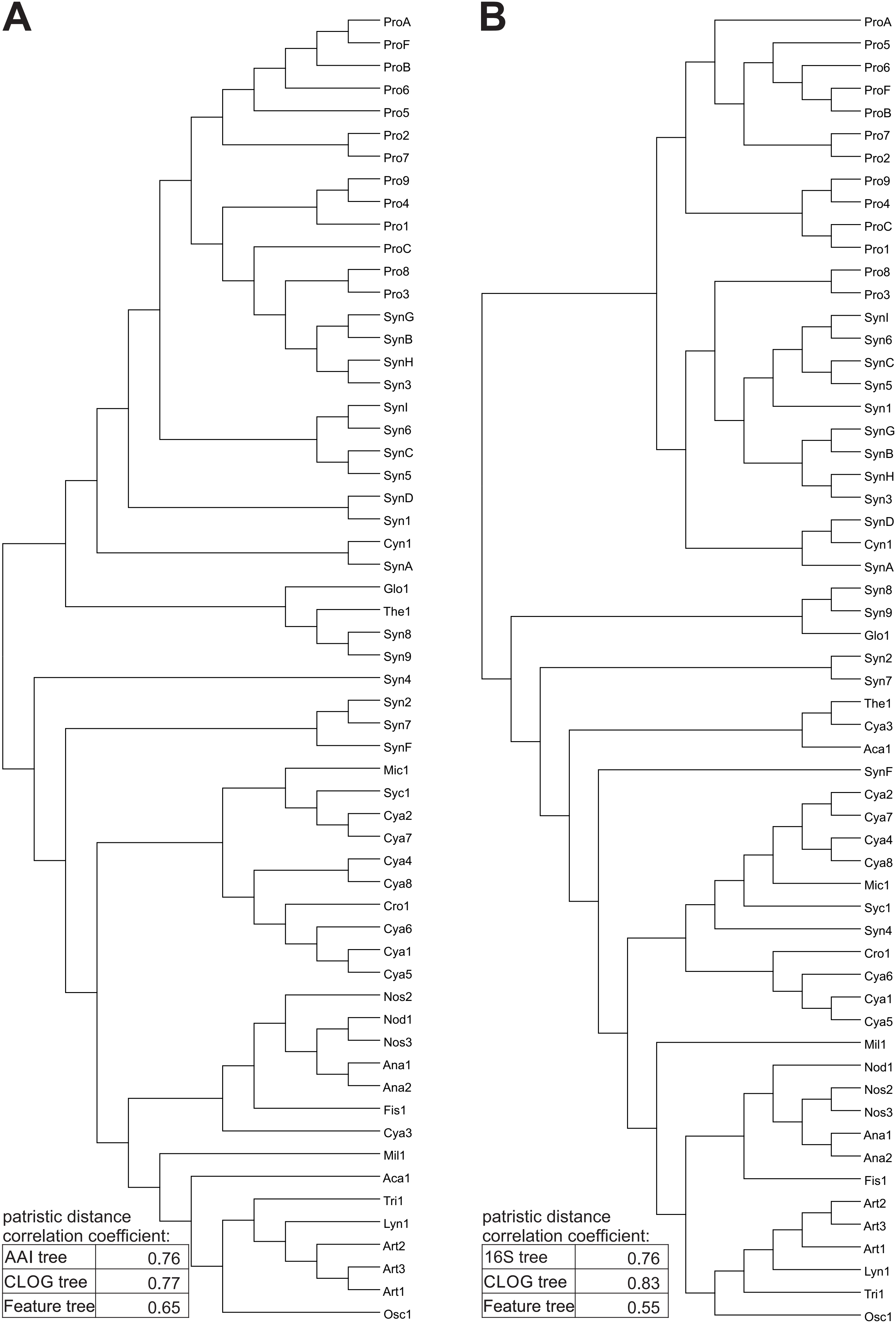
A
ProA
ProF
ProB
Pro6
Pro5
Pro2
Pro7
Pro9
Pro4
Pro1
ProC
Pro8
Pro3
SynG
SynB
SynH
Syn3
SynI
Syn6
SynC
Syn5
SynD
Syn1
Cyn1
SynA
Glo1
The1
Syn8
Syn9
Syn4
Syn2
Syn7
SynF
Mic1
Syc1
Cya2
Cya7
Cya4
Cya8
Cro1
Cya6
Cya1
Cya5
Nos2
Nod1
Nos3
Ana1
Ana2
Fis1
Cya3
Mil1
Aca1
Tri1
Lyn1
Art2
Art3
Art1
Osc1
patristic distance
correlation coefficient:
AAI tree 0.76
CLOG tree 0.77
Feature tree 0.65
B
ProA
Pro5
Pro6
ProF
ProB
Pro7
Pro2
Pro9
Pro4
ProC
Pro1
Pro8
Pro3
SynI
Syn6
SynC
Syn5
Syn1
SynG
SynB
SynH
Syn3
SynD
Cyn1
SynA
Syn8
Syn9
Glo1
Syn2
Syn7
The1
Cya3
Aca1
SynF
Cya2
Cya7
Cya4
Cya8
Mic1
Syc1
Syn4
Cro1
Cya6
Cya1
Cya5
Mil1
Nod1
Nos2
Nos3
Ana1
Ana2
Fis1
Art2
Art3
Art1
Lyn1
Tri1
Osc1
patristic distance
correlation coefficient:
16S tree 0.76
CLOG tree 0.83
Feature tree 0.55
