# Supplementary material for: The composition of the global and feature specific cyanobacterial core-genomes
Source: Front Microbiol. 2015 Mar 19;6:219. doi: 10.3389/fmicb.2015.00219 (PMC4365693; doi:10.3389/fmicb.2015.00219)
Supplement: Supplementary file 1 [file DataSheet1.ZIP › AddFiles/File 7.PDF]

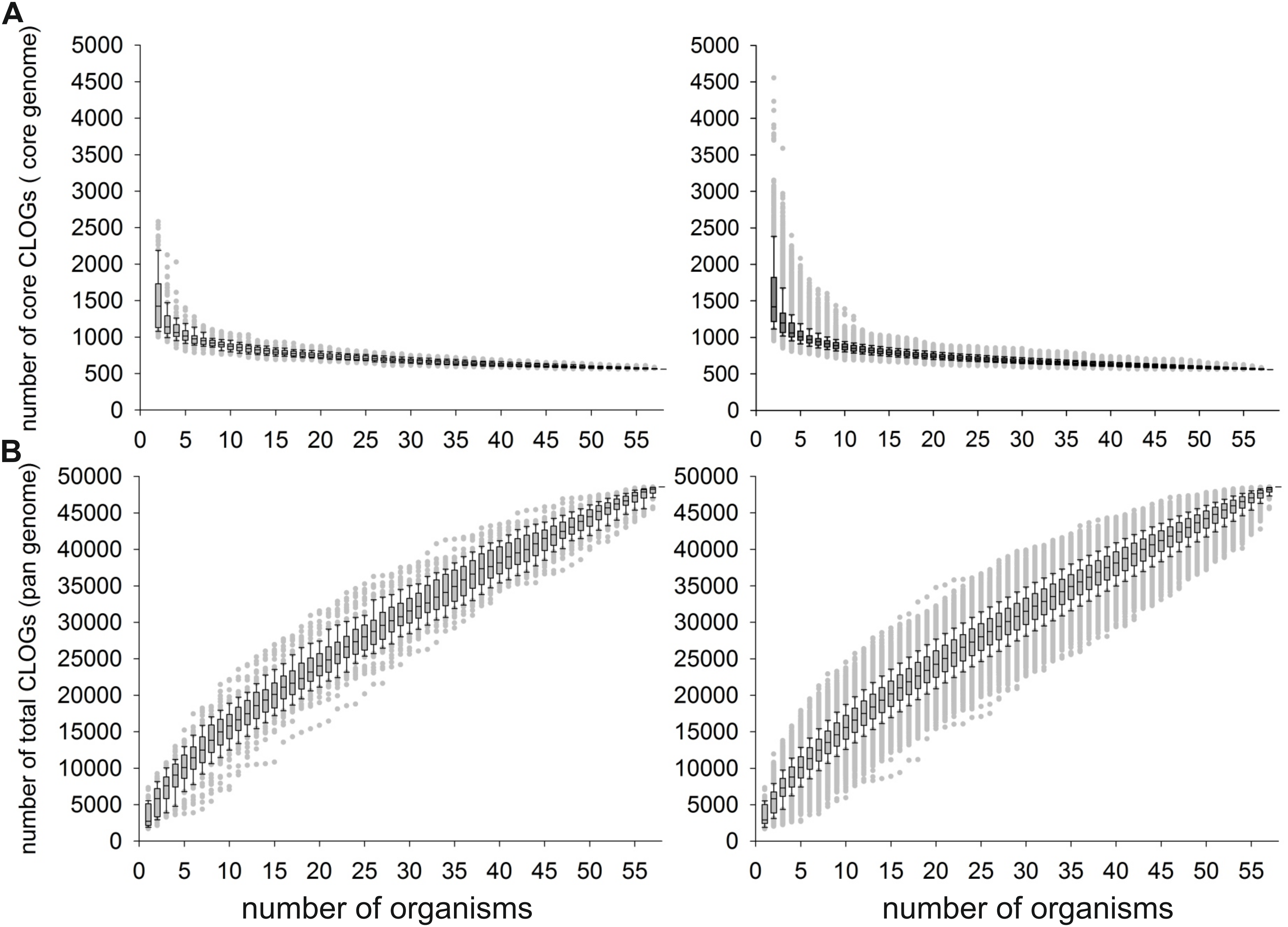

A
B
number of core CLOGs (core genome)
number of total CLOGs (pan genome)
number of organisms
number of organisms
