# Supplementary material for: The composition of the global and feature specific cyanobacterial core-genomes
Source: Front Microbiol. 2015 Mar 19;6:219. doi: 10.3389/fmicb.2015.00219 (PMC4365693; doi:10.3389/fmicb.2015.00219)
Supplement: Supplementary file 1 [file DataSheet1.ZIP › AddFiles/File 8.DOCX]

| **Additional file 8: Distribution of *Anabaena* sp. PCC 7120 proteins involved in oxidative phosphorylation and photosynthesis in the core-genomes of the different clades of the feature based tree.**  In total 153 genes of *Anabaena* sp. PCC 7120 have been assigned to the two KEGG categories, but only 63 have been identified in the CORE-GENOME of cyanobacteria, most likely because *Gloeobacter violaceus* PCC 7421 does not contain the thylakoid system. This notion is consistent with the occurrence of 50% of all proteins assigned to oxidative phosphorylation, but only 36% of the proteins assigned to photosynthesis to the CORE-GENOME. It is further supported by the large number of 35 photosynthetic proteins (38% of all photosynthetic proteins assigned) to be encoded by the core genome with root of clade I (separating Glo1 from Ana1) or not to be assigned to any core genome (category: *Anabaena* sp. PCC 7120). Moreover, in total 98 of the 153 genes (65%) were assigned as energy production and conversion genes by COG, while of the genes assigned to the core-genome with root of clades V, IV and III (Figure 4) 92% were detected by the KEGG and COG approach.  In the subsequent table the columns are: the root of the clade of the feature based tree for which the core genome was defined (column 1), the KEGG number of the protein (column 2), the name of the protein (column 3), the accession number of the according gene in *Anabaena* sp. PCC 7120 (column 4) and the functional category according to KEGG (column 6) and the functional category according to COG (column 7: Energy prod…. energy production and conversion; none… no functional assignment in COG, other…a functional assignment distinct from energy production and conversion) | | | | | |
| --- | --- | --- | --- | --- | --- |
| **Root of the clade** | **KEGG** | **NAME** | **Acc** | **Process according to KEGG** | **Process according to COG** |
| **V** | K02113 | ATPF1D | all0006 | Oxidative phosphorylation | Energy prod. |
|  | K02108 | ATPF0A | all0010 | Oxidative phosphorylation | Energy prod. |
|  | K02111 | ATPF1A | all0005 | Oxidative phosphorylation | Energy prod. |
|  | K02110 | ATPF0C | asl0009 | Oxidative phosphorylation | Energy prod. |
|  | K02115 | ATPF1G | all0004 | Oxidative phosphorylation | Energy prod. |
|  | K05572 | ndhA | alr0223 | Oxidative phosphorylation | Energy prod. |
|  | K05574 | ndhC | all3842 | Oxidative phosphorylation | Energy prod. |
|  | K05576 | ndhE | alr0226 | Oxidative phosphorylation | Energy prod. |
|  | K05578 | ndhG | alr0225 | Oxidative phosphorylation | Energy prod. |
|  | K05579 | ndhH | alr3355 | Oxidative phosphorylation | Energy prod. |
|  | K02109 | ATPF0B | all0007 | Oxidative phosphorylation | Energy prod. |
|  | K02109 | ATPF0B | all0008 | Oxidative phosphorylation | Energy prod. |
|  | K02274 | coxA | alr0951 | Oxidative phosphorylation | Energy prod. |
|  | K02274 | coxA | alr2515 | Oxidative phosphorylation | Energy prod. |
|  | K02274 | coxA | alr2732 | Oxidative phosphorylation | Energy prod. |
|  | K05577 | ndhF | alr3956 | Oxidative phosphorylation | Energy prod. |
|  | K02276 | coxC | alr0952 | Oxidative phosphorylation | Energy prod. |
|  | K02276 | coxC | alr2516 | Oxidative phosphorylation | Energy prod. |
|  | K02275 | coxB | alr0950 | Oxidative phosphorylation | Energy prod. |
|  | K02275 | coxB | alr2514 | Oxidative phosphorylation | Energy prod. |
|  | K05575 | ndhD | alr0348 | Oxidative phosphorylation | Energy prod. |
|  | K05575 | ndhD | alr3957 | Oxidative phosphorylation | Energy prod. |
|  | K05575 | ndhD | alr5050 | Oxidative phosphorylation | Energy prod. |
|  | K03885 | ndh | alr4094 | Oxidative phosphorylation | Energy prod. |
|  | K05585 | ndhN | alr4216 | Oxidative phosphorylation | none |
|  | K02301 | cyoE | all0948 | Oxidative phosphorylation | Other |
|  | K02259 | COX15 | all0949 | Oxidative phosphorylation | Other |
|  | K00937 | ppk | alr3593 | Oxidative phosphorylation | Other |
|  | K02635 | petB | alr3421 | Photosynthesis | Energy prod. |
|  | K02108 | ATPF0A, | all0010 | Photosynthesis | Energy prod. |
|  | K02109 | ATPF0B, | all0008 | Photosynthesis | Energy prod. |
|  | K02109 | ATPF0B, | all0007 | Photosynthesis | Energy prod. |
|  | K02637 | petD | alr3422 | Photosynthesis | Energy prod. |
|  | K02115 | ATPF1G, | all0004 | Photosynthesis | Energy prod. |
|  | K02113 | ATPF1D, | all0006 | Photosynthesis | Energy prod. |
|  | K02110 | ATPF0C, | asl0009 | Photosynthesis | Energy prod. |
|  | K02111 | ATPF1A, | all0005 | Photosynthesis | Energy prod. |
|  | K02639 | petF | all4148 | Photosynthesis | Energy prod. |
|  | K02639 | petF | alr0784 | Photosynthesis | Energy prod. |
|  | K02639 | petF | all2919 | Photosynthesis | Energy prod. |
|  | K02634 | petA | all2452 | Photosynthesis | Energy prod. |
|  | K02693 | psaE | asr4319 | Photosynthesis | Energy prod. |
|  | K02690 | psaB | alr5314 | Photosynthesis | Energy prod. |
|  | K02690 | psaB | alr5155 | Photosynthesis | Energy prod. |
|  | K02694 | psaF | all0109 | Photosynthesis | Energy prod. |
|  | K02703 | psbA | alr3742 | Photosynthesis | Energy prod. |
|  | K02703 | psbA | alr4592 | Photosynthesis | Energy prod. |
|  | K02703 | psbA | alr4866 | Photosynthesis | Energy prod. |
|  | K02703 | psbA | all3572 | Photosynthesis | Energy prod. |
|  | K02703 | psbA | alr3727 | Photosynthesis | Energy prod. |
|  | K02706 | psbD | alr4548 | Photosynthesis | Energy prod. |
|  | K02706 | psbD | alr4290 | Photosynthesis | Energy prod. |
|  | K02707 | psbE | asr3845 | Photosynthesis | Energy prod. |
|  | K02704 | psbB | all0138 | Photosynthesis | Energy prod. |
|  | K02705 | psbC | all4002 | Photosynthesis | Energy prod. |
|  | K02705 | psbC | all4003 | Photosynthesis | Energy prod. |
|  | K02705 | psbC | all4000 | Photosynthesis | Energy prod. |
|  | K02705 | psbC | alr4291 | Photosynthesis | Energy prod. |
|  | K02709 | psbH | asl0846 | Photosynthesis | Energy prod. |
|  | K02689 | psaA | alr5154 | Photosynthesis | Energy prod. |
|  | K02717 | psbP | all3076 | Photosynthesis | Energy prod. |
|  | K02716 | psbO | all3854 | Photosynthesis | Energy prod. |
|  | K02699 | psaL | all0107 | Photosynthesis | Energy prod. |
| **IV** | K05573 | ndhB | all4883 | Oxidative phosphorylation | Energy prod. |
|  | K00239 | sdhA, | all2970 | Oxidative phosphorylation | Energy prod. |
|  | K01507 | ppa | all3570 | Oxidative phosphorylation | Energy prod. |
|  | K05581 | ndhJ | all3840 | Oxidative phosphorylation | Energy prod. |
|  | K05577 | ndhF | alr0869 | Oxidative phosphorylation | Energy prod. |
|  | K05577 | ndhF | alr4156 | Oxidative phosphorylation | Energy prod. |
|  | K05575 | ndhD | alr0870 | Oxidative phosphorylation | Energy prod. |
|  | K05582 | ndhK | all3841 | Oxidative phosphorylation | Energy prod. |
|  | K02692 | psaD | all0329 | Photosynthesis | none |
|  | K02720 | psbV | all0259 | Photosynthesis | none |
|  | K08906 | petJ | alr4251 | Photosynthesis | Energy prod. |
|  | K08906 | petJ | all0161 | Photosynthesis | Energy prod. |
|  | K08906 | petJ | asl0256 | Photosynthesis | none |
|  | K02636 | petC | all2453 | Photosynthesis | Energy prod. |
|  | K02636 | petC | all0606 | Photosynthesis | Energy prod. |
|  | K02636 | petC | all1512 | Photosynthesis | Energy prod. |
|  | K02636 | petC | all4511 | Photosynthesis | Energy prod. |
|  | K02289 | cpcF | alr0533 | Photosynthesis - antenna proteins | Energy prod. |
|  | K02288 | cpcE | alr0532 | Photosynthesis - antenna proteins | Energy prod. |
|  | K02631 | pecE | alr0526 | Photosynthesis - antenna proteins | Energy prod. |
| **III** | K00241 | sdhC | all3341 | Oxidative phosphorylation | Energy prod. |
|  | K00240 | sdhB | all0945 | Oxidative phosphorylation | Energy prod. |
| **II** | K05583 | ndhL | asr4809 | Oxidative phosphorylation | none |
|  | K02276 | coxC | alr2734 | Oxidative phosphorylation | Energy prod. |
|  | K08902 | psb27 | all1258 | Photosynthesis | none |
|  | K02697 | psaJ | asl0108 | Photosynthesis | none |
|  | K02697 | psaJ | asl3190 | Photosynthesis | none |
|  | K02719 | psbU | alr1216 | Photosynthesis | none |
|  | K02096 | apcE | alr0020 | Photosynthesis - antenna proteins | none |
|  | K02094 | apcC | asr0023 | Photosynthesis - antenna proteins | none |
|  | K02284 | cpcA | alr0529 | Photosynthesis - antenna proteins | none |
|  | K02286 | cpcC | alr0530 | Photosynthesis - antenna proteins | none |
|  | K02097 | apcF | all2327 | Photosynthesis - antenna proteins | none |
|  | K02290 | cpcG | alr0534 | Photosynthesis - antenna proteins | none |
|  | K02290 | cpcG | alr0535 | Photosynthesis - antenna proteins | none |
|  | K02290 | cpcG | alr0536 | Photosynthesis - antenna proteins | none |
|  | K02290 | cpcG | alr0537 | Photosynthesis - antenna proteins | none |
| **I** | K05584 | ndhM | all1732 | Oxidative phosphorylation | none |
|  | K02114 | ATPF1E | all5038 | Oxidative phosphorylation | energy prod |
|  | K02112 | ATPF1B | all5039 | Oxidative phosphorylation | energy prod |
|  | K02275 | coxB | alr2731 | Oxidative phosphorylation | energy prod |
|  | K05575 | ndhD | alr4157 | Oxidative phosphorylation | energy prod |
|  | K03885 | ndh | all1126 | Oxidative phosphorylation | energy prod |
|  | K03885 | ndh | all2964 | Oxidative phosphorylation | energy prod |
|  | K03885 | ndh | alr5211 | Oxidative phosphorylation | energy prod |
|  | K03885 | ndh | all1127 | Oxidative phosphorylation | energy prod |
|  | K03885 | ndh | all1553 | Oxidative phosphorylation | energy prod |
|  | K02724 | psbZ | asr3992 | Photosynthesis | none |
|  | K02112 | ATPF1B, | all5039 | Photosynthesis | energy prod |
|  | K02691 | psaC | asr3463 | Photosynthesis | energy prod |
|  | K02114 | ATPF1E, | all5038 | Photosynthesis | energy prod |
|  | K08903 | psb28 | all0801 | Photosynthesis | none |
|  | K02711 | psbJ | asr3848 | Photosynthesis | none |
|  | K02710 | psbI | asr1277 | Photosynthesis | none |
|  | K02713 | psbL | asr3847 | Photosynthesis | none |
|  | K03689 | petN | asl4263 | Photosynthesis | none |
|  | K02702 | psaX | asr1283 | Photosynthesis | none |
|  | K02700 | psaM | asr4657 | Photosynthesis | none |
|  | K02643 | petM | asl4754 | Photosynthesis | none |
|  | K02708 | psbF | asr3846 | Photosynthesis | none |
|  | K02698 | psaK | alr5290 | Photosynthesis | none |
|  | K02698 | psaK | asr4775 | Photosynthesis | none |
|  | K02698 | psaK | asr5289 | Photosynthesis | none |
|  | K02722 | psbX | asr0941 | Photosynthesis | none |
|  | K02714 | psbM | asl0883 | Photosynthesis | none |
|  | K02641 | petH | all4121 | Photosynthesis | other |
|  | K02095 | apcD | all3653 | Photosynthesis - antenna proteins | none |
|  | K02092 | apcA | alr0021 | Photosynthesis - antenna proteins | none |
|  | K02092 | apcA | all0450 | Photosynthesis - antenna proteins | none |
|  | K02093 | apcB | alr0022 | Photosynthesis - antenna proteins | none |
|  | K02287 | cpcD | asr0531 | Photosynthesis - antenna proteins | none |
| **Specific for Anabaena sp.** | K05587 | hoxF | alr0752 | Oxidative phosphorylation | energy prod |
|  | K05586 | hoxE | alr0751 | Oxidative phosphorylation | energy prod |
|  | K05588 | hoxU | alr0762 | Oxidative phosphorylation | energy prod |
|  | K05580 | ndhI | alr0224 | Oxidative phosphorylation | energy prod |
|  | K00425 | cydA | all4024 | Oxidative phosphorylation | energy prod |
|  | K00426 | cydB | all4023 | Oxidative phosphorylation | energy prod |
|  | K02638 | petE | all0258 | Photosynthesis | energy prod |
|  | K02718 | psbT | asl0137 | Photosynthesis | none |
|  | K02723 | psbY | asr1025 | Photosynthesis | none |
|  | K02640 | petG | asr1366 | Photosynthesis | none |
|  | K02712 | psbK | asl0885 | Photosynthesis | none |
|  | K08904 | psb28-2 | all1082 | Photosynthesis | none |
|  | K02696 | psaI | asl3849 | Photosynthesis | none |
|  | K02642 | petL | asl1922 | Photosynthesis | none |
|  | K02285 | cpcB | alr0528 | Photosynthesis - antenna proteins | none |
|  | K02632 | pecF | alr0527 | Photosynthesis - antenna proteins | none |
|  | K02629 | pecB | alr0523 | Photosynthesis - antenna proteins | none |
|  | K02628 | pecA | alr0524 | Photosynthesis - antenna proteins | none |
|  | K02630 | pecC | alr0525 | Photosynthesis - antenna proteins | none |
