# Supplementary material for: The composition of the global and feature specific cyanobacterial core-genomes
Source: Front Microbiol. 2015 Mar 19;6:219. doi: 10.3389/fmicb.2015.00219 (PMC4365693; doi:10.3389/fmicb.2015.00219)
Supplement: Supplementary file 1 [file DataSheet1.ZIP › AddFiles/File 9.DOCX]

**Additional file 9: Functional categories in pairwise shared CLOG splits exemplified on *Anabaena* sp. PCC 7120**

| **Functional category** | **Functional process** | **Root of the clade for which the core-genome is defined** | | | | | |
| --- | --- | --- | --- | --- | --- | --- | --- |
|  |  | **A** | **B** | **C** | **D** | **E** | **F** |
| **Information storage and processing** | **J** | 7 | 17 | 4 | 12 | 8 | 90 |
|  | **K** | 17 | 16 | 2 | 7 | 5 | 11 |
|  | **L** | 17 | 23 | 3 | 7 | 6 | 37 |
|  | **KL** | 1 | - | - | 1 | - | 3 |
|  | **JKL** | - | - | 1 | - | - | - |
|  | **Total** | **42** | **56** | **10** | **27** | **19** | **141** |
| **Cellular processes and signaling** | **D** | 3 | 9 | 3 | 4 | 1 | 11 |
|  | **M** | 34 | 55 | 13 | 27 | 22 | 27 |
|  | **N** | - | - | - | 1 | - | - |
|  | **O** | 9 | 40 | 7 | 15 | 8 | 40 |
|  | **T** | 29 | 41 | 14 | 14 | 2 | 8 |
|  | **U** | 2 | 12 | 4 | 4 | - | 10 |
|  | **V** | 15 | 30 | 1 | 5 | 4 | 5 |
|  | **MU** | - | - | - | - | 1 | 1 |
|  | **NT** | 1 | 3 | 2 | 2 | - | - |
|  | **NU** | 3 | 3 | 3 | 1 | 6 | - |
|  | **OU** | - | 2 | 1 | 2 | - | 1 |
|  | **NOU** | - | - | - | - | 1 | - |
|  | **Total** | **96** | **195** | **48** | **75** | **45** | **103** |
| **Metabolism** | **C** | 21 | 41 | 6 | 17 | 17 | 45 |
|  | **E** | 16 | 34 | 11 | 17 | 7 | 49 |
|  | **F** | 4 | 10 | 1 | 2 | 8 | 23 |
|  | **G** | 14 | 41 | 8 | 9 | 6 | 22 |
|  | **H** | 15 | 22 | 7 | 4 | 16 | 46 |
|  | **I** | 4 | 9 | - | 3 | 5 | 15 |
|  | **P** | 36 | 63 | 3 | 8 | 3 | 13 |
|  | **Q** | 13 | 23 | - | 1 | - | 3 |
|  | **CE** | - | - | - | - | - | 1 |
|  | **CH** | 1 | 1 | 1 | - | - | - |
|  | **CP** | 1 | - | - | - | 2 | 1 |
|  | **CQ** | - | 1 | - | 1 | 2 | - |
|  | **EF** | - | - | - | - | - | 3 |
|  | **EG** |  | - | - | - | - | 1 |
|  | **EH** | 1 | 3 | - | 1 | - | 5 |
|  | **EP** | 2 | - | 2 | 1 | 1 | - |
|  | **EQ** | - | - | - | 2 | - | - |
|  | **FP** | - | - | - | - | - | 1 |
|  | **HI** | - | - | - | - | - | 1 |
|  | **HP** | 1 | - | - | - | - | - |
|  | **HQ** | - | 1 | - | - | - | - |
|  | **IQ** | - | 3 | - | - | - | 2 |
|  | **PQ** | - | - | - | 1 | - | - |
|  | **Total** | **129** | **252** | **39** | **67** | **67** | **231** |
| **Poorly characterized** | **R** | 46 | 78 | 41 | 50 | 26 | 35 |
|  | **S** | 67 | 102 | 19 | 33 | 16 | 24 |
|  | **mixed processes** | 23 | 38 | 18 | 13 | 8 | 17 |
|  | **-----** | 433 | 298 | 49 | 88 | 41 | 53 |
|  | **Total** | **569** | **516** | **127** | **184** | **91** | **129** |
| Shown is the functional category (column 1), the abbreviation of the COG of the functional process (Table 2, column 3) and the number of sequences of *Anabaena* PCC 7120 assigned to the different clade core-genomes (columns 3-8) based on the pairwise shared CLOGs tree (Figure 3 A). Please note, the numbers are accumulative: e.g. in E only the number of genes found in addition to the number found in F are shown. | | | | | | | |
