# Supplementary figures and images for: The composition of the global and feature specific cyanobacterial core-genomes
Source: Front Microbiol. 2015 Mar 19;6:219. doi: 10.3389/fmicb.2015.00219 (PMC4365693; doi:10.3389/fmicb.2015.00219)

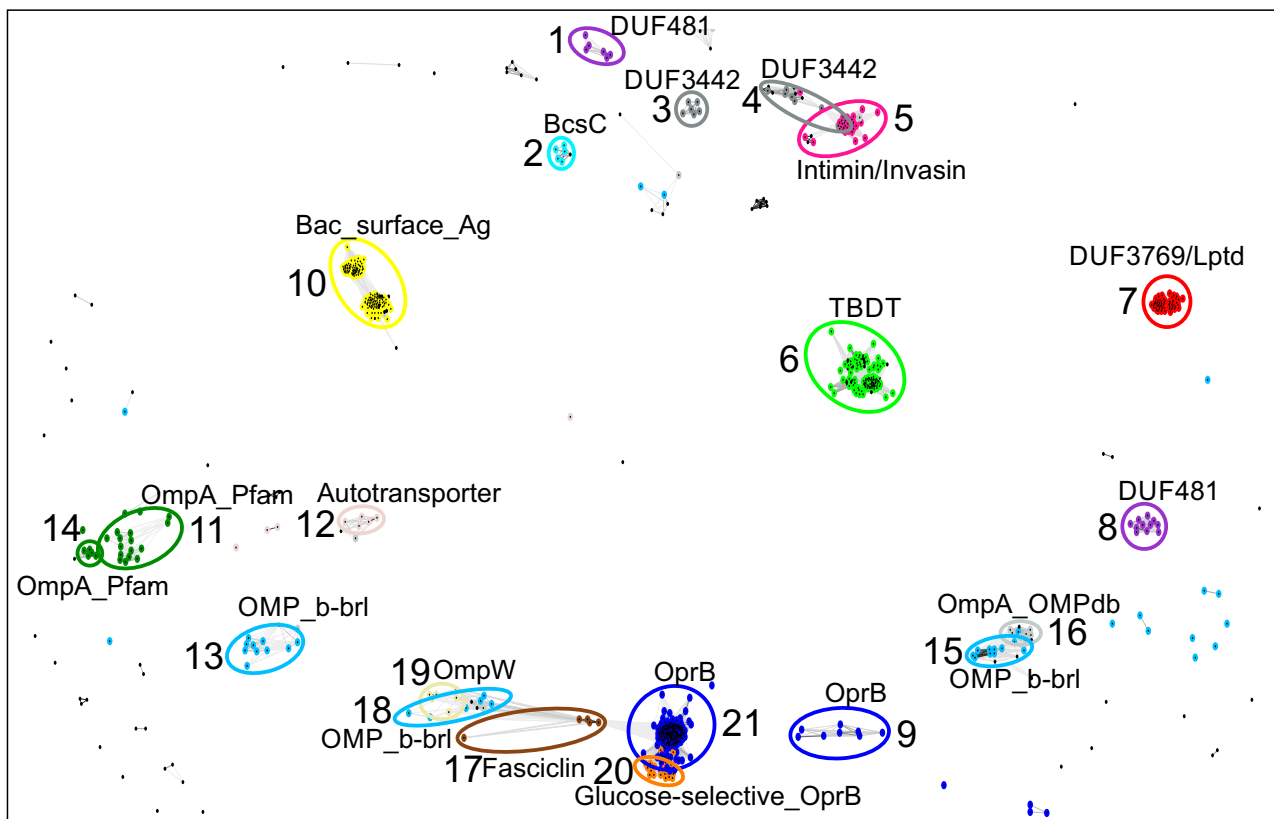

Supplement: Supplementary file 1 [file DataSheet1.ZIP › AddFiles/File 12.PDF]

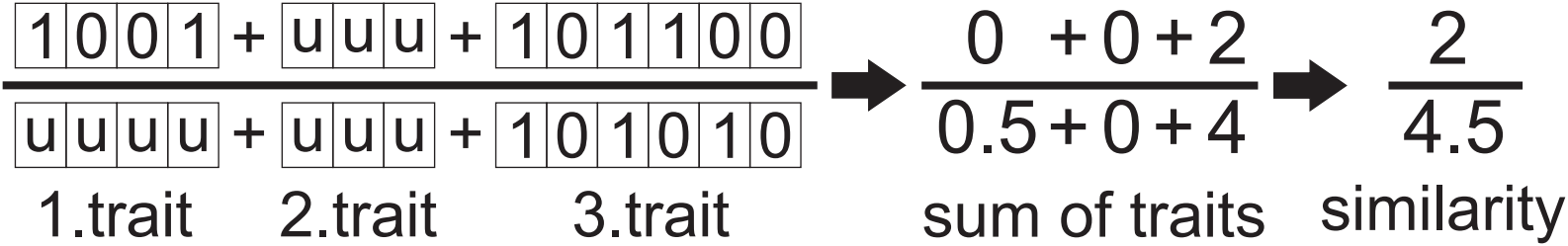

Supplement: Supplementary file 1 [file DataSheet1.ZIP › AddFiles/File 2.PDF]

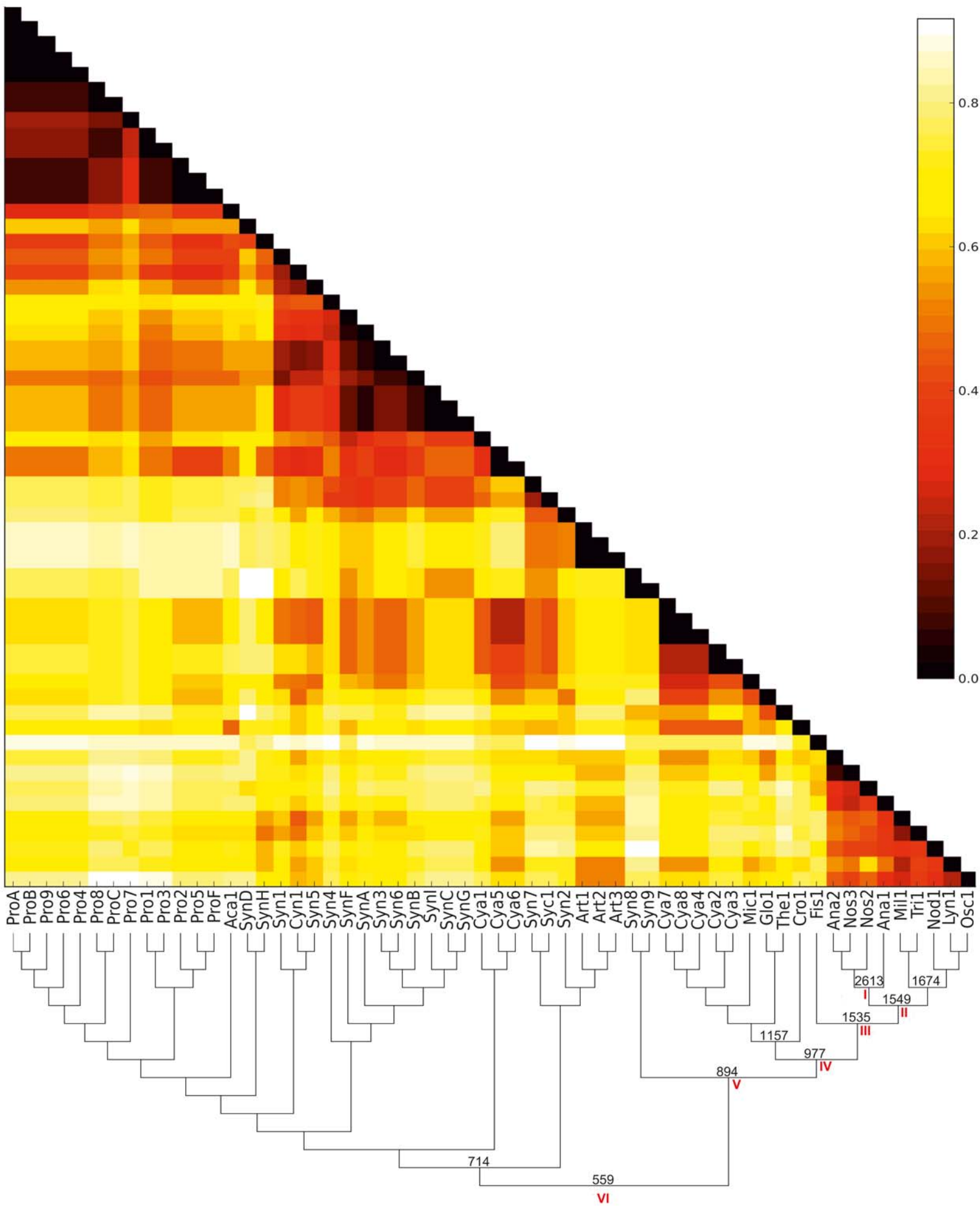

Supplement: Supplementary file 1 [file DataSheet1.ZIP › AddFiles/File 3.PDF]

**A**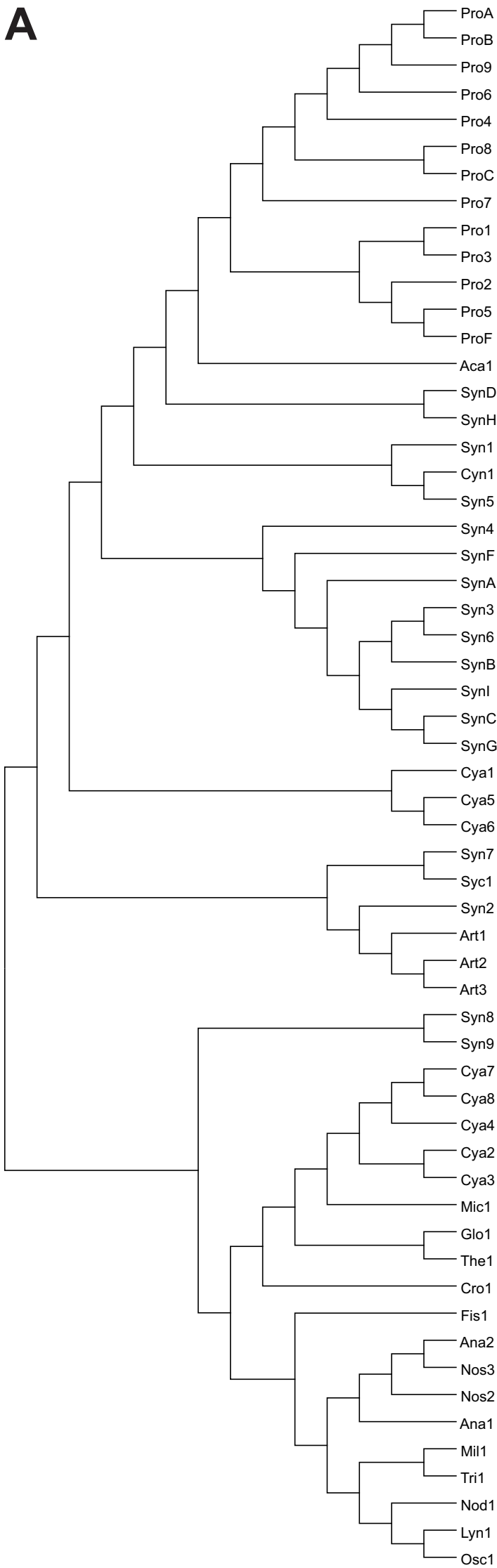**B**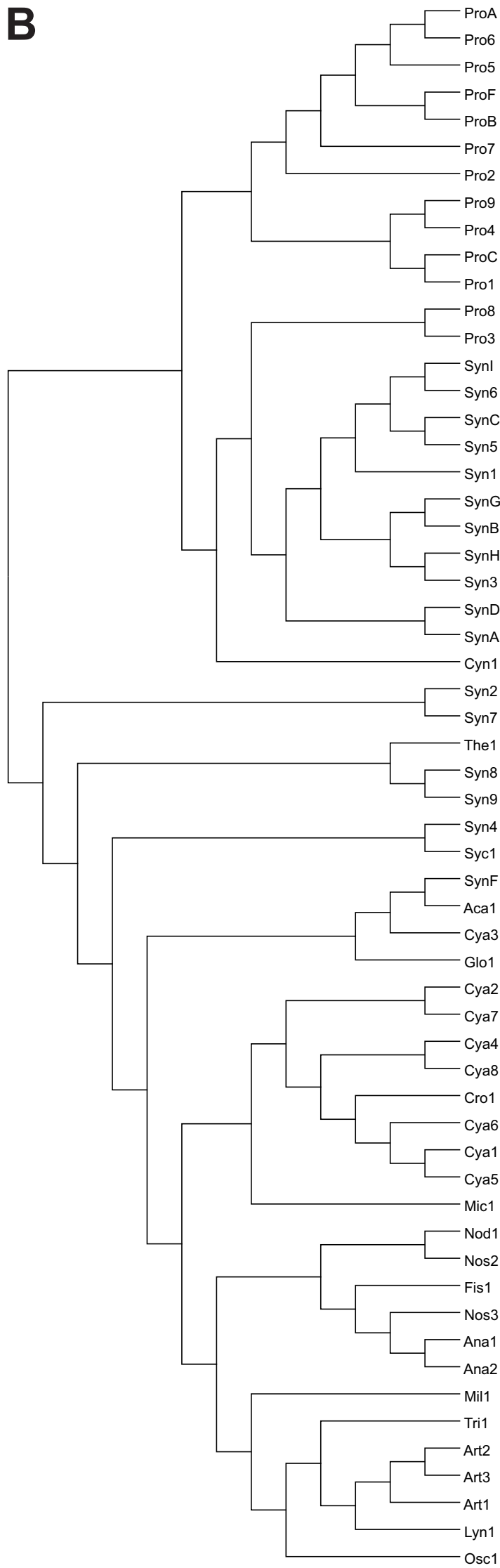

Supplement: Supplementary file 1 [file DataSheet1.ZIP › AddFiles/File 4.PDF]
